# Supplementary material for: Synthesis and Antiproliferative Effect of 3,4,5-Trimethoxylated Chalcones on Colorectal and Prostatic Cancer Cells
Source: Pharmaceuticals (Basel). 2024 Sep 13;17(9):1207. doi: 10.3390/ph17091207 (PMC11434868; doi:10.3390/ph17091207)
Supplement: Supplementary file 1 [file pharmaceuticals-17-01207-s001.zip › pharmaceuticals-3173706-supplementary.pdf]

Supplementary materials

# Synthesis and antiproliferative effect of 3,4,5-trimethoxylated chalcones on colorectal and prostatic cancer cells

**Cécile Letulle [1,2], François-Xavier Toublet [1], Aline Pinon [1], Soufyane Hba [1,6], Aurélie Laurent [1], Vincent Sol [1], Catherine Fagnère [1], Benjamin Rioux [1], Florent Allais [3], Sophie Michallet [4], Laurence Lafanechère [4], Youness Limami [5,6], Mounia Oudghiri [6], Mohamed Othman [2], Adam Daïch [2], Bertrand Liagre [1], Ata Martin Lawson [2,*] and Christelle Pouget [1,*]**

[1] Univ. Limoges, LABC*i*S, UR 22722, Faculty of Pharmacy, F-87000, Limoges, France; cecile.letulle@etu.univ-lehavre.fr (C.L.); francois-xavier.toublet@unilim.fr (F.-X.T.); aline.pinon@unilim.fr (A.P.); hbasoufyane@gmail.com (S.H.) ; aurelie.laurent@unilim.fr (A.L.) ; vincent.sol@unilim.fr (V.S.); catherine.fagnere@unilim.fr (C.F.); benjamin.rioux@cma-nouvelleaquitaine.fr (B.R.); bertrand.liagre@unilim.fr (B.L.)

[2] Normandie Univ, UNILEHAVRE, URCOM, UR 3221, INC3M, FR 3038 CNRS, F-76600 Le Havre, France; mohamed.othman@univ-lehavre.fr (M.O.); adam.daich@univ-lehavre.fr (A.D.);

[3] URD Agro-Biotechnologies Industrielles (ABI), CEBB (Centre Européen de Biotechnologie et de Bioéconomie), AgroParisTech, F-51110 Pomacle, France; florent.allais@agroparistech.fr (F.A.) ;

[4] Univ. Grenoble Alpes, Institute for Advanced Biosciences, Team Cytoskeletal Dynamics and Nuclear Functions, INSERM U1209, CNRS UMR5309, F-38000 Grenoble, France; sophie.michallet@univ-grenoble-alpes.fr (S.M.); laurence.lafanechere@univ-grenoble-alpes.fr (L.L.);

[5] Hassan First University of Settat, Laboratory of Health Sciences and Technologies, Higher Institute of Health Sciences, Settat 26000, Morocco; youness.limami@gmail.com (Y.L.);

[6] Hassan II University, Laboratory of Immunology and Biodiversity, Faculty of Sciences Ain Chock, B.P. 2693, Maarif, Casablanca 20100, Morocco; mouniaoudghiri@gmail.com (M.Ou.)

* Correspondence: christelle.pouget@unilim.fr (C.P.); ata-fmartin.lawson@univ-lehavre.fr (A.M.L.)

Total number of Pages: 27 (Pages S1-S27).
Total number of Figures: 46 (Figures S1-S46).
Total number of Protocols: 2 (Protocols S1-S2).

## Table of contents

**Citation:** To be added by editorial staff during production.

Academic Editor: Firstname Lastname

Received: date
Revised: date
Accepted: date
Published: date

**Copyright:** © 2024 by the authors. Submitted for possible open access publication under the terms and conditions of the Creative Commons Attribution (CC BY) license (https://creativecommons.org/licenses/by/4.0/).

NMR analysis ........ 3
Figure S1: $^{1}$H spectrum of chalcone 1 ........ 4
Figure S2: $^{13}$C spectrum of chalcone 1 ........ 4
Figure S3: $^{1}$H spectrum of chalcone 2 ........ 5
Figure S4: $^{13}$C spectrum of chalcone 2 ........ 5
Figure S5: $^{1}$H spectrum of chalcone 3 ........ 6
Figure S6: $^{13}$C spectrum of chalcone 3 ........ 6
Figure S7: $^{1}$H spectrum of chalcone 4 ........ 7
Figure S8: $^{13}$C spectrum of chalcone 4 ........ 7
Figure S9: $^{1}$H spectrum of chalcone 5 ........ 8
Figure S10: $^{13}$C spectrum of chalcone 5 ........ 8
Figure S11: $^{1}$H spectrum of chalcone 6 ........ 9

Figure S12: $^{13}$C spectrum of chalcone 6 ... 9
Figure S13: $^{1}$H spectrum of chalcone 7 ... 10
Figure S14: $^{13}$C spectrum of chalcone 7 ... 10
Figure S15: $^{1}$H spectrum of chalcone 8 ... 11
Figure S16: $^{13}$C spectrum of chalcone 8 ... 11
Figure S17: $^{1}$H spectrum of chalcone 9 ... 12
Figure S18: $^{13}$C spectrum of chalcone 9 ... 12
Figure S19: $^{1}$H spectrum of chalcone 10 ... 13
Figure S20: $^{13}$C spectrum of chalcone 10 ... 13
Figure S21: $^{1}$H spectrum of chalcone 11 ... 14
Figure S22: $^{13}$C spectrum of chalcone 11 ... 15
Figure S23: $^{1}$H spectrum of chalcone 12 ... 15
Figure S24: $^{13}$C spectrum of chalcone 12 ... 16
Figure S25: $^{1}$H spectrum of chalcone 13 ... 16
Figure S26: $^{13}$C spectrum of chalcone 13 ... 17
Figure S27: $^{1}$H spectrum of chalcone 14 ... 17
Figure S28: $^{13}$C spectrum of chalcone 14 ... 18
Figure S29: $^{1}$H spectrum of chalcone 15 ... 18
Figure S30: $^{13}$C spectrum of chalcone 15 ... 19
HRMS analysis ... 19
Figure S31. HRMS spectrum of chalcone 1 ... 19
Figure S32. HRMS spectrum of chalcone 2 ... 19
Figure S33. HRMS spectrum of chalcone 3 ... 20
Figure S34. HRMS spectrum of chalcone 4 ... 20
Figure S35. HRMS spectrum of chalcone 5 ... 20
Figure S36. HRMS spectrum of chalcone 6 ... 21
Figure S37. HRMS spectrum of chalcone 7 ... 21
Figure S38. HRMS spectrum of chalcone 10 ... 21
Figure S39. HRMS spectrum of chalcone 11 ... 21
Figure S40. HRMS spectrum of chalcone 12 ... 22
Figure S41. HRMS spectrum of chalcone 13 ... 23
Figure S42. HRMS spectrum of chalcone 14 ... 23
Figure S43. HRMS spectrum of chalcone 15 ... 23
Protocols ... 24
Protocol S1. Synthesis of 6-oxo-1,6-dihydropyridine-3-carbaldehyde: ... 24
Protocol S2. General procedure for the synthesis of 4-oxo-4*H*-chromene-3-carbaldehydes: ... 24
Cell viability ... 25

Figure S44. Effect of chalcone 11 on cell viability in human CRC (HCT116 and HT-29) and human PCa (DU145 and PC3) cells at 24 and 48 h. ........ 25

Trypan blue dye exclusion method ........ 26

Figure S45. Cell count at 48 h (viable and dead cells) relative to the concentration of compounds 10 and 13 for human colorectal (HCT116 and HT-29) cancer cells. ........ 26

Figure S46. Cell count at 48 h (viable and dead cells) relative to the concentration of compounds 10 and 13 for human prostatic (DU145 and PC3) cancer cells. ........ 27

## NMR analysis

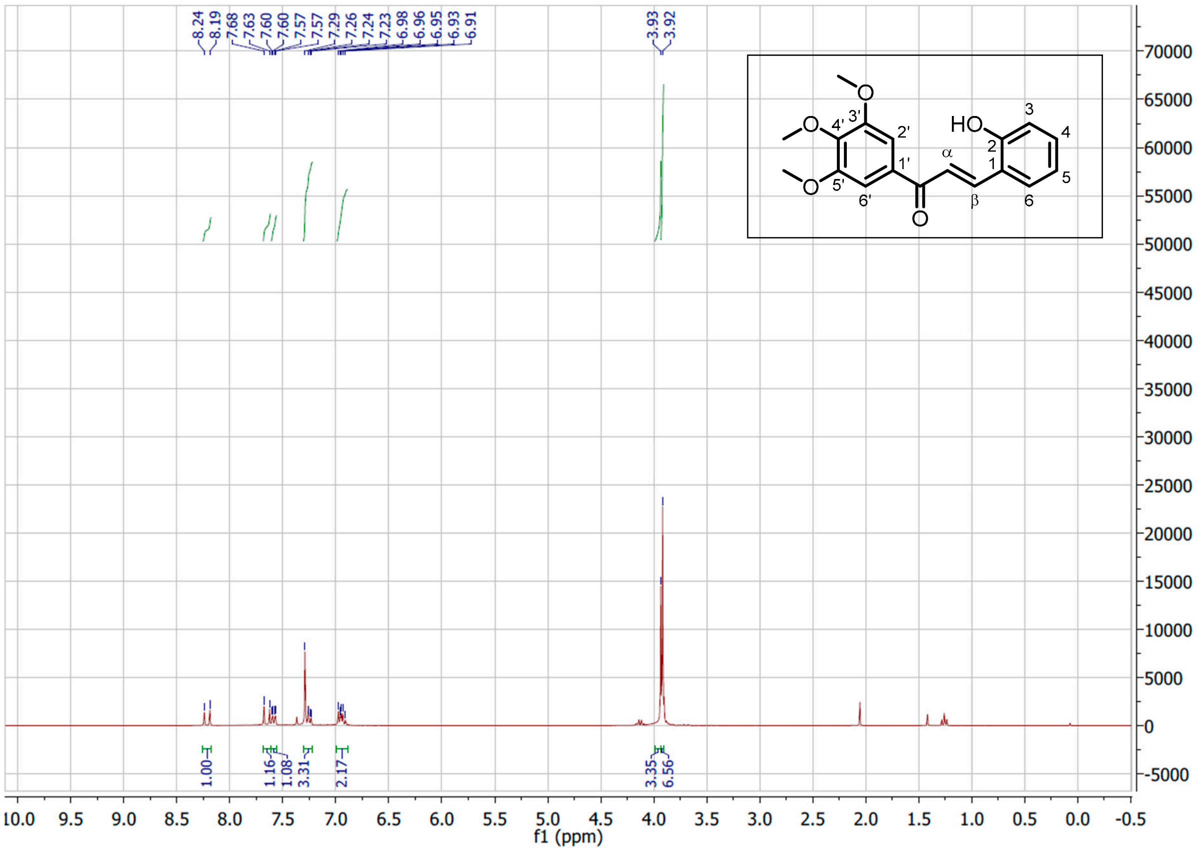

## Figure S1: $^{1}$H spectrum of chalcone 1

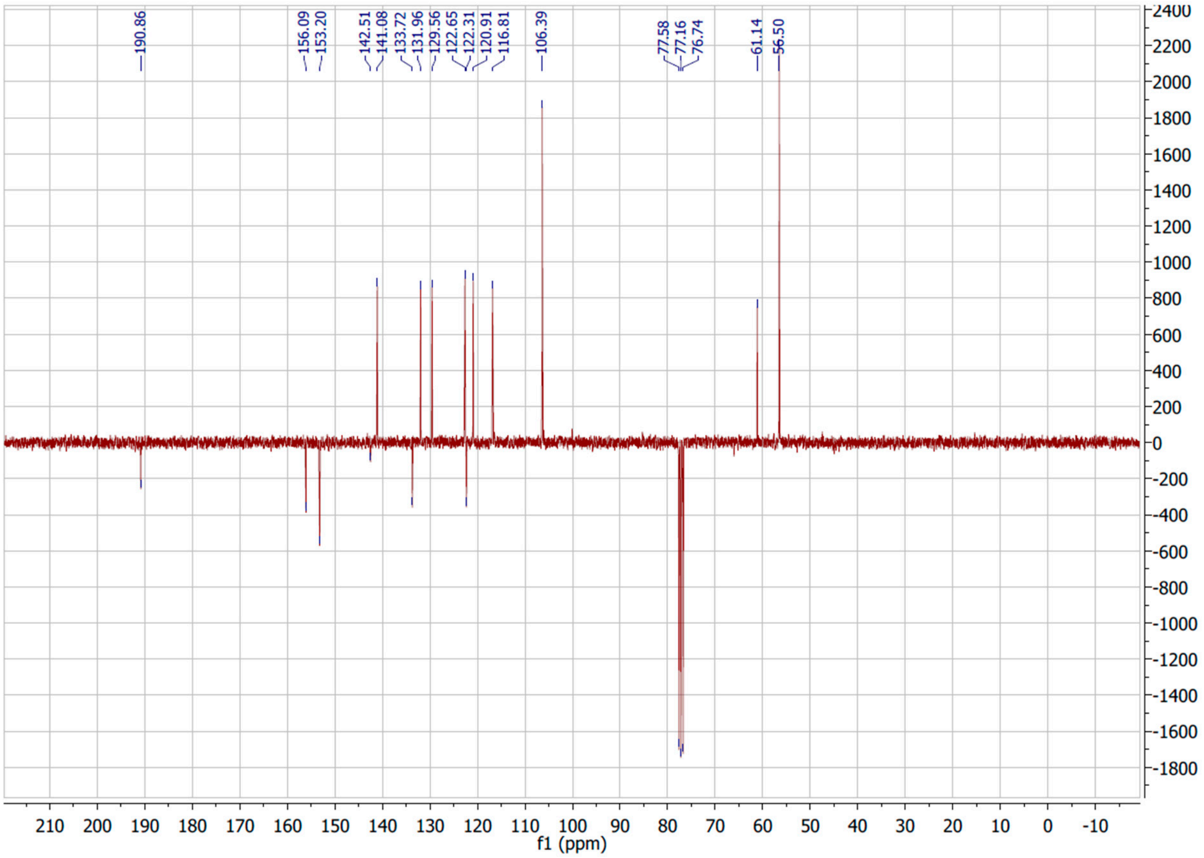


## Figure S2: $^{13}$C spectrum of chalcone 1

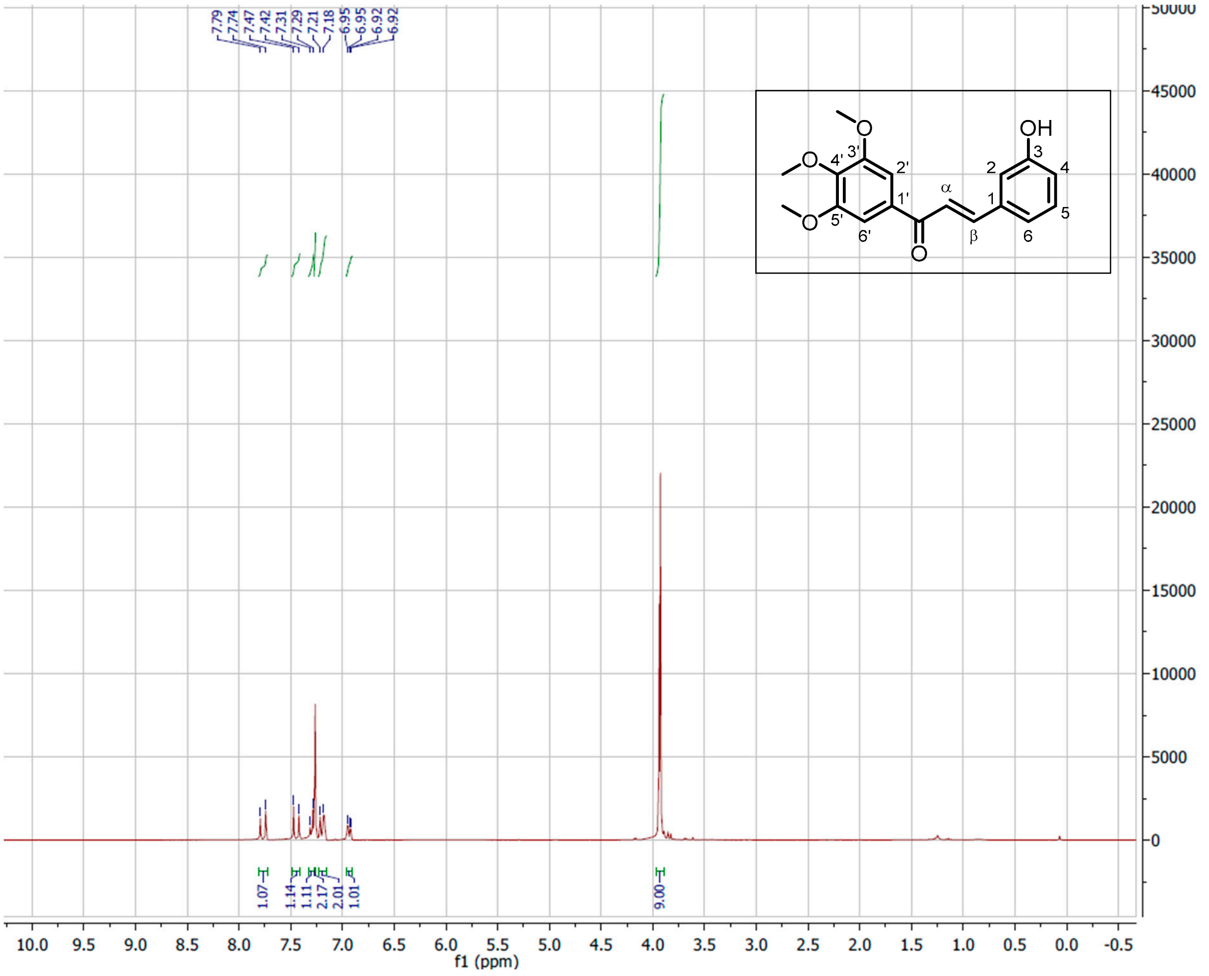

## Figure S3: $^{1}H$ spectrum of chalcone 2

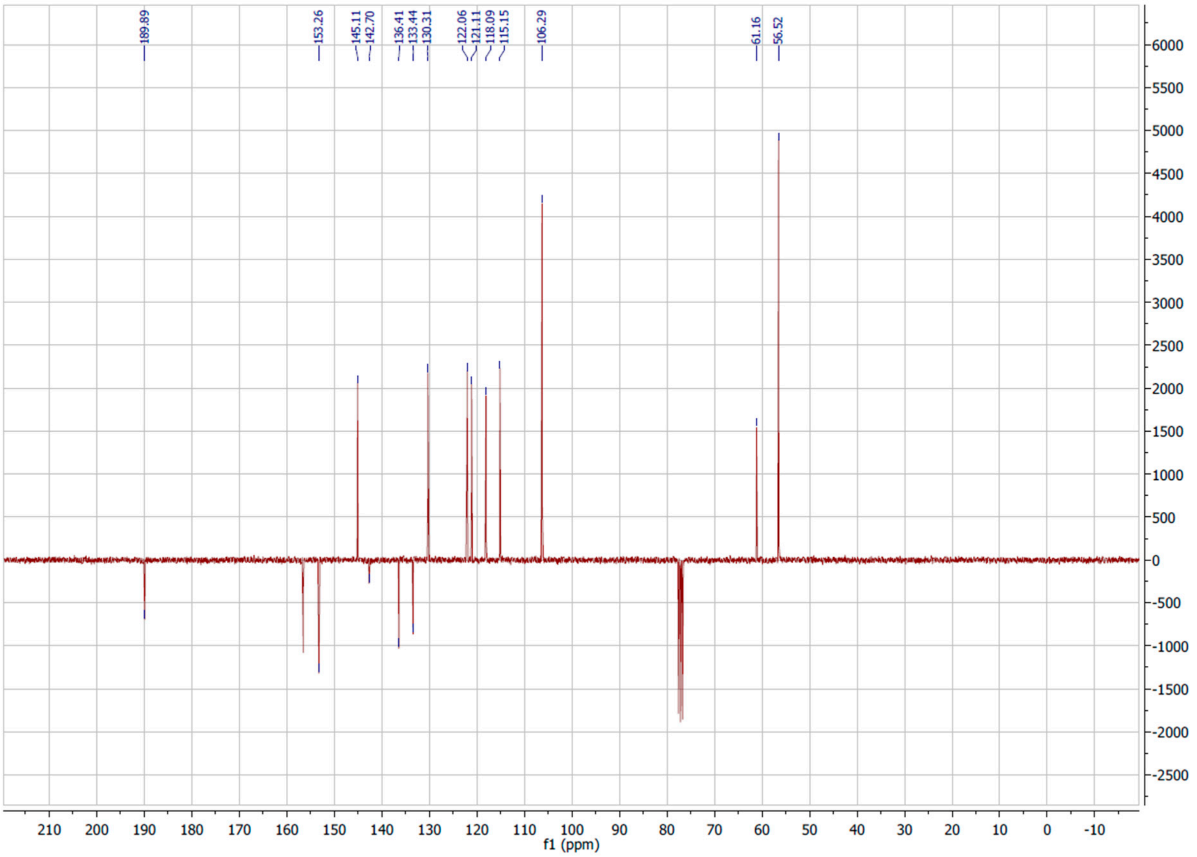


## Figure S4: $^{13}C$ spectrum of chalcone 2

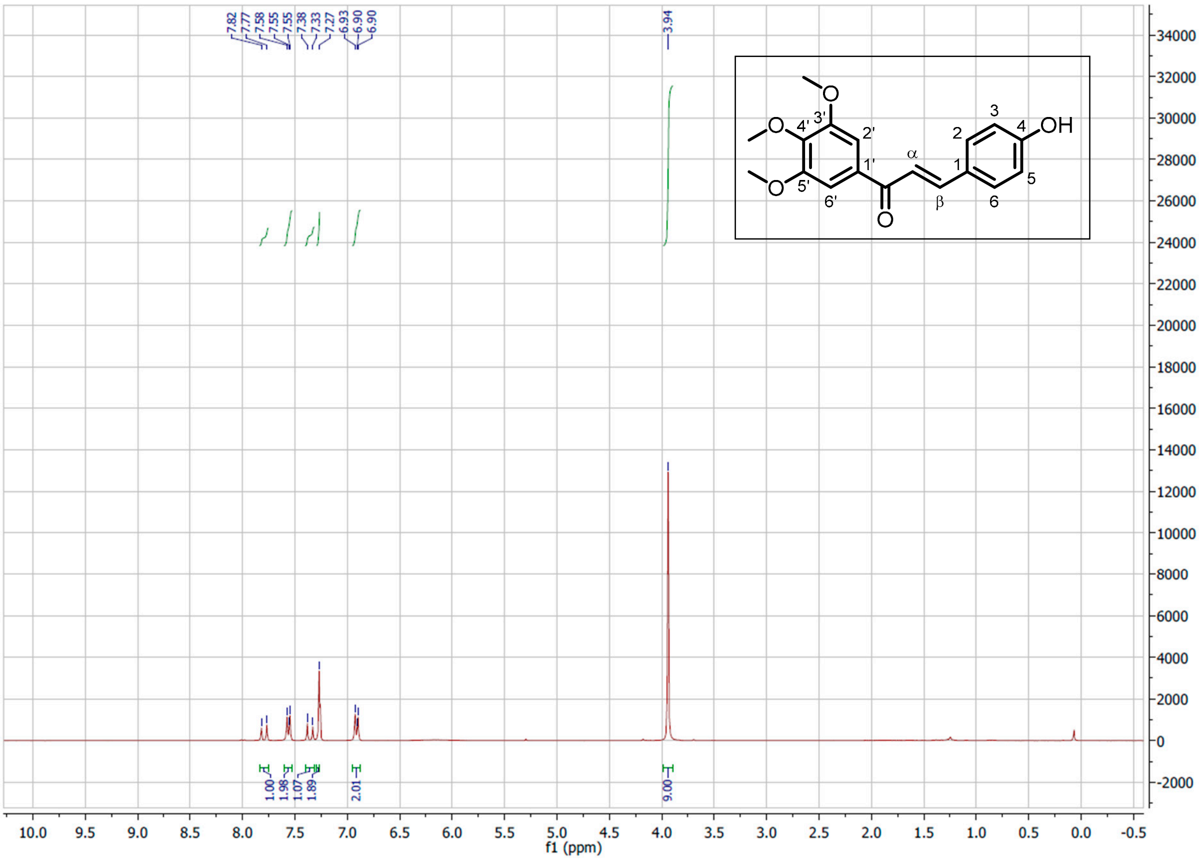

**Figure S5: $^{1}$H spectrum of chalcone 3**

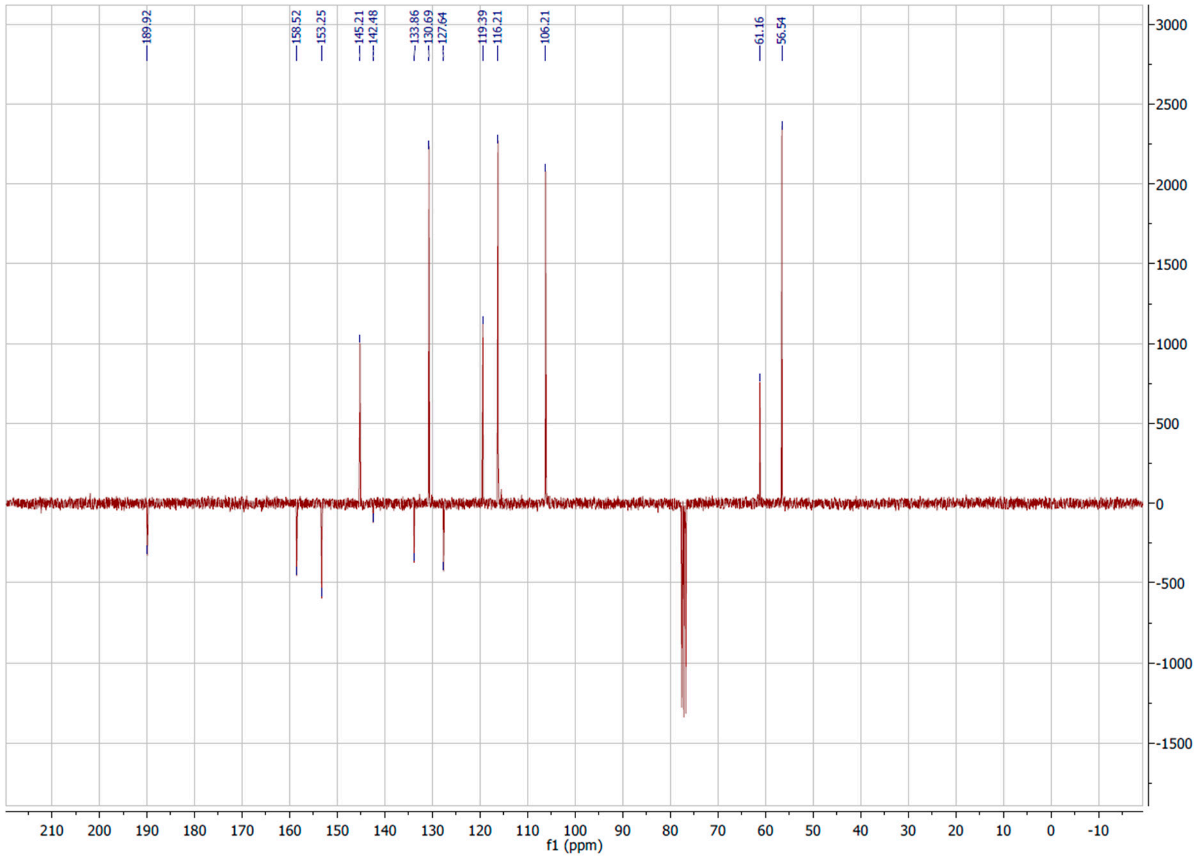

**Figure S6: $^{13}$C spectrum of chalcone 3**

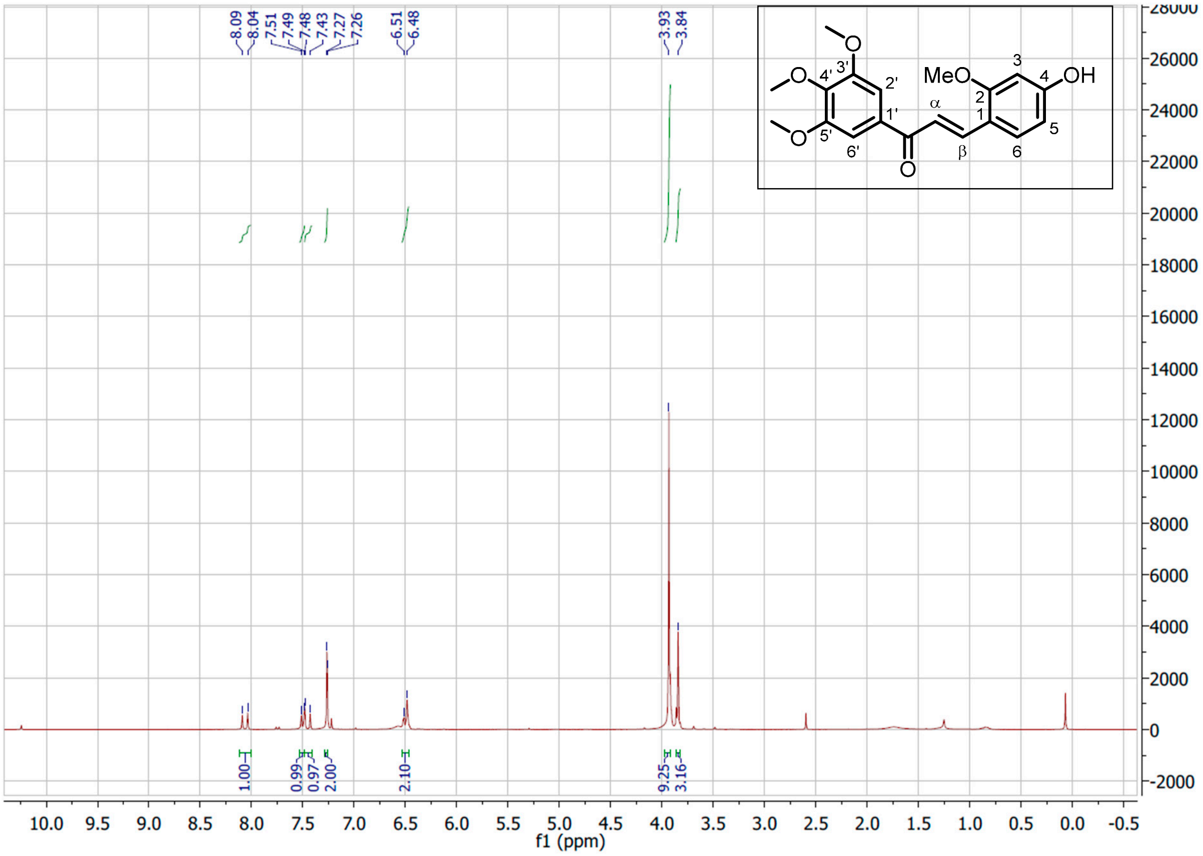

Figure S7: $^{1}$H spectrum of chalcone 4

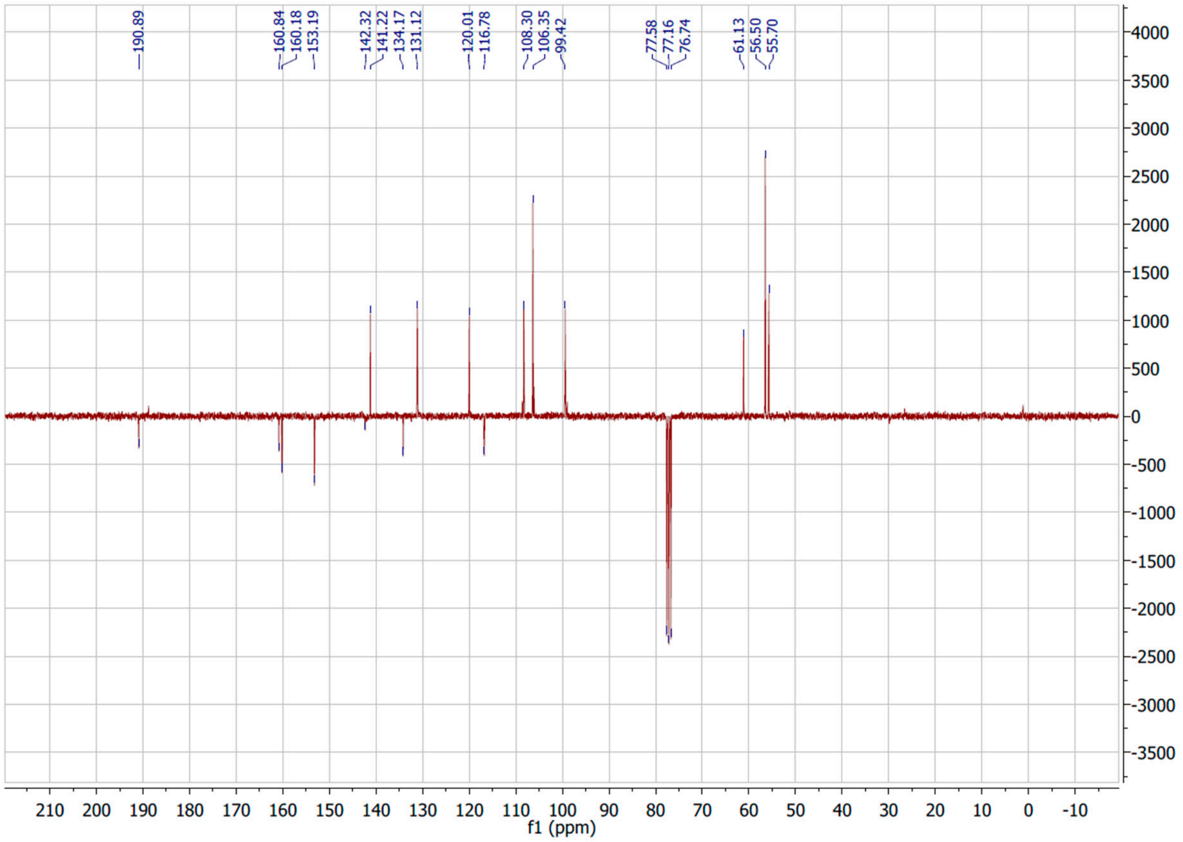

Figure S8: $^{13}$C spectrum of chalcone 4

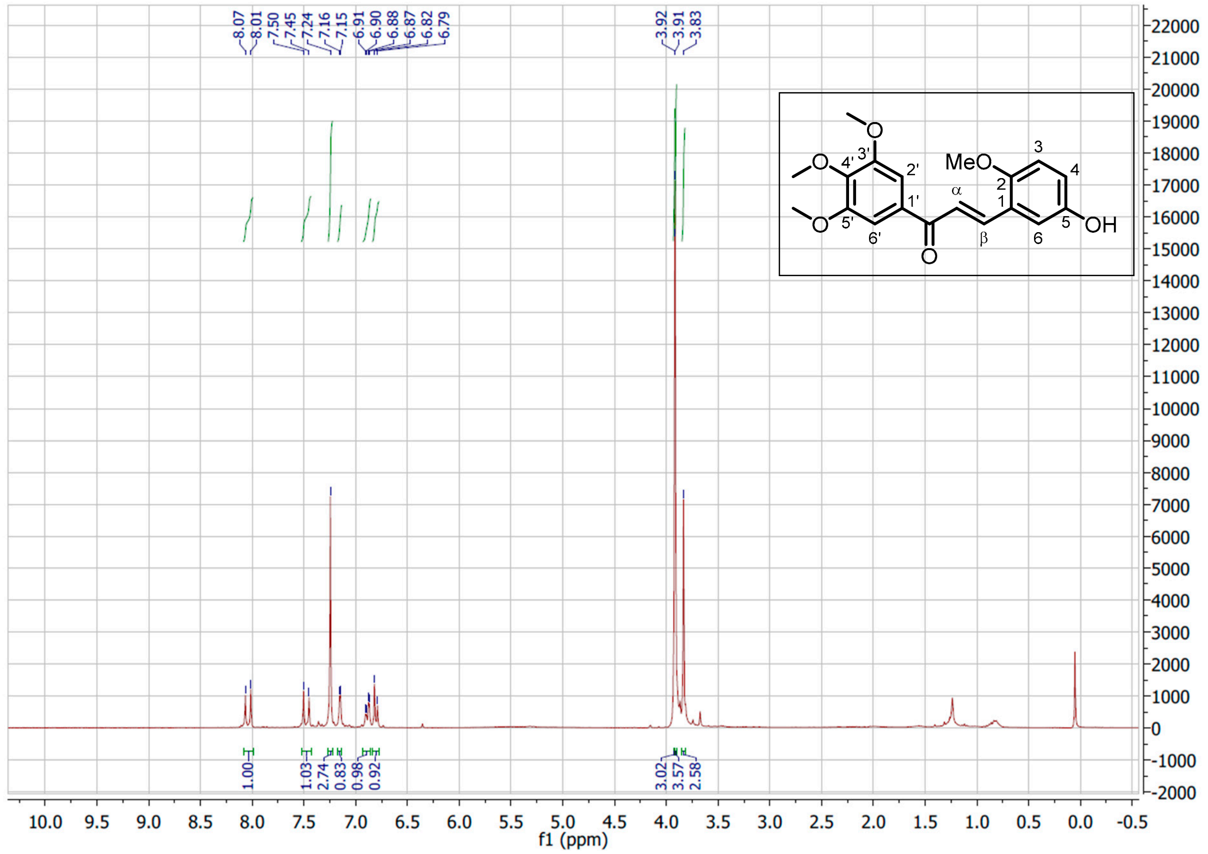

**Figure S9: $^{1}$H spectrum of chalcone 5**

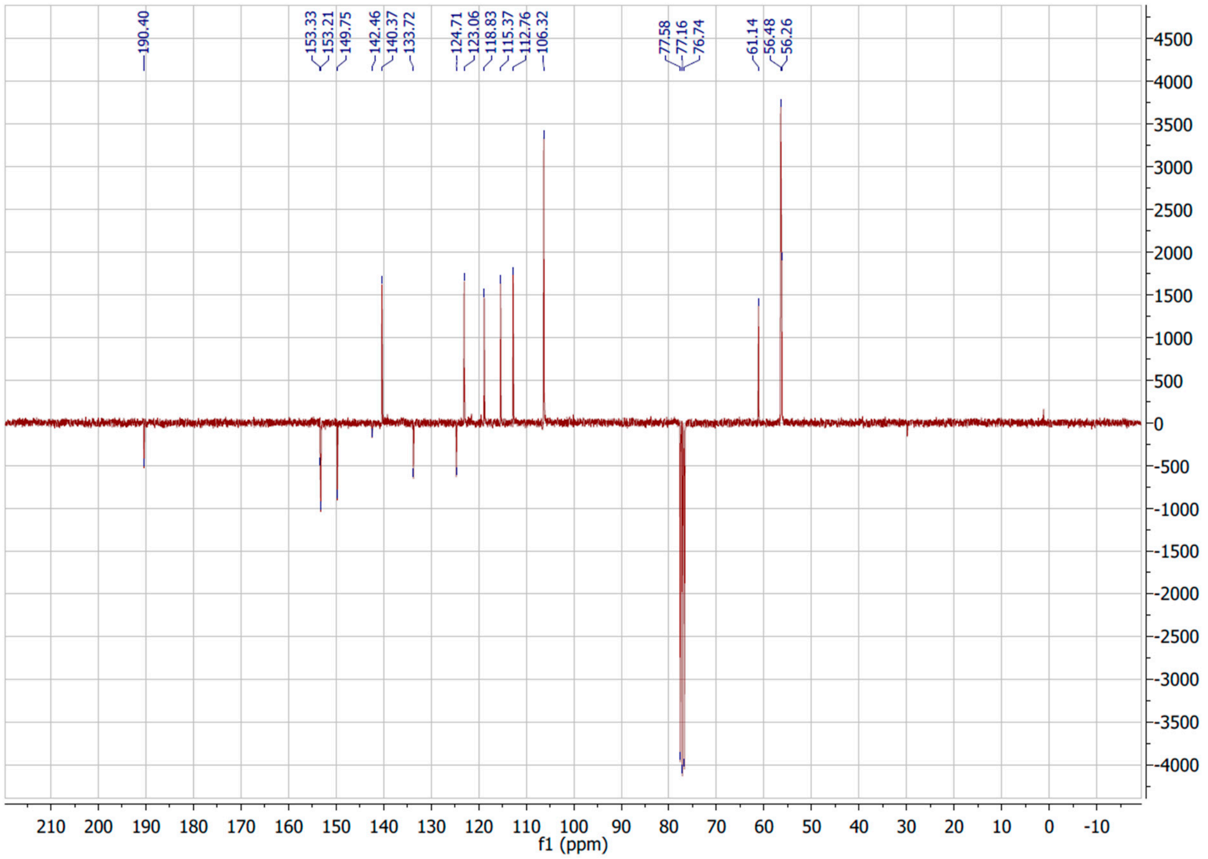

**Figure S10: $^{13}$C spectrum of chalcone 5**

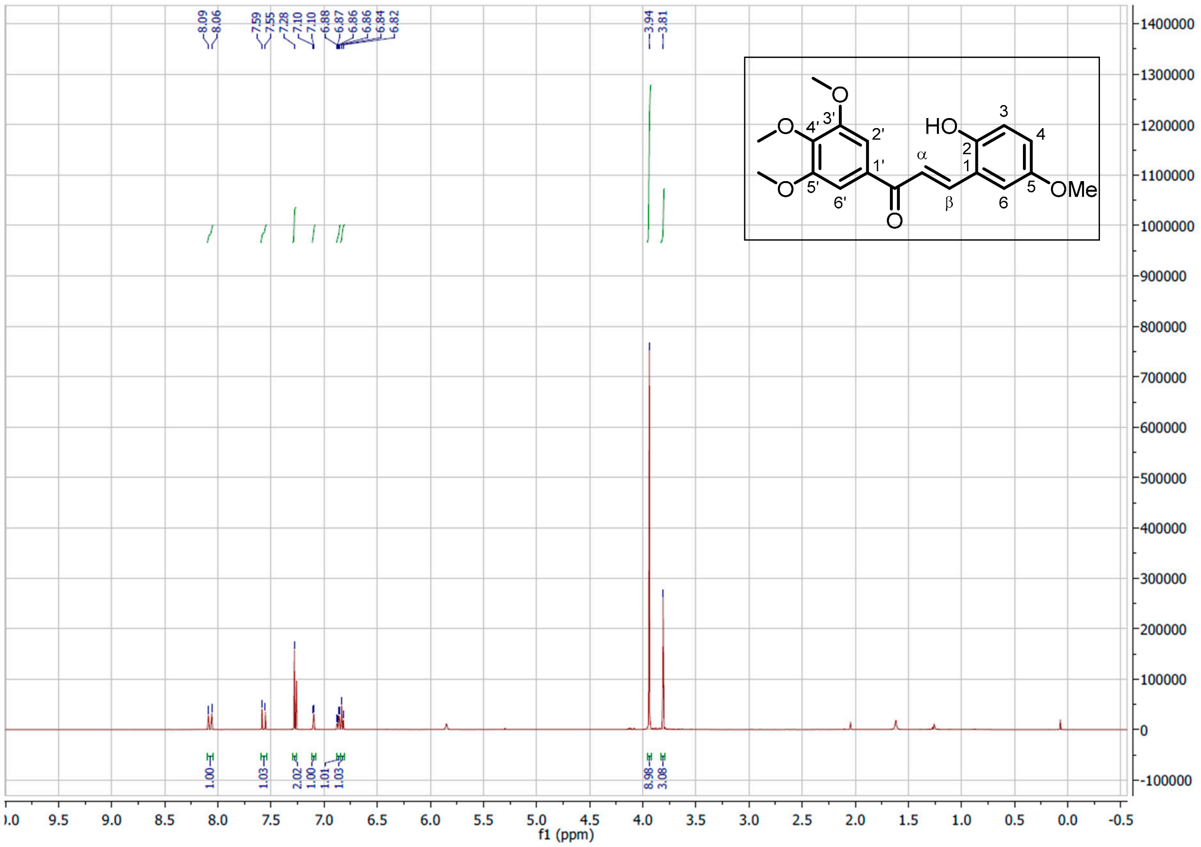

**Figure S11: $^{1}$H spectrum of chalcone 6**

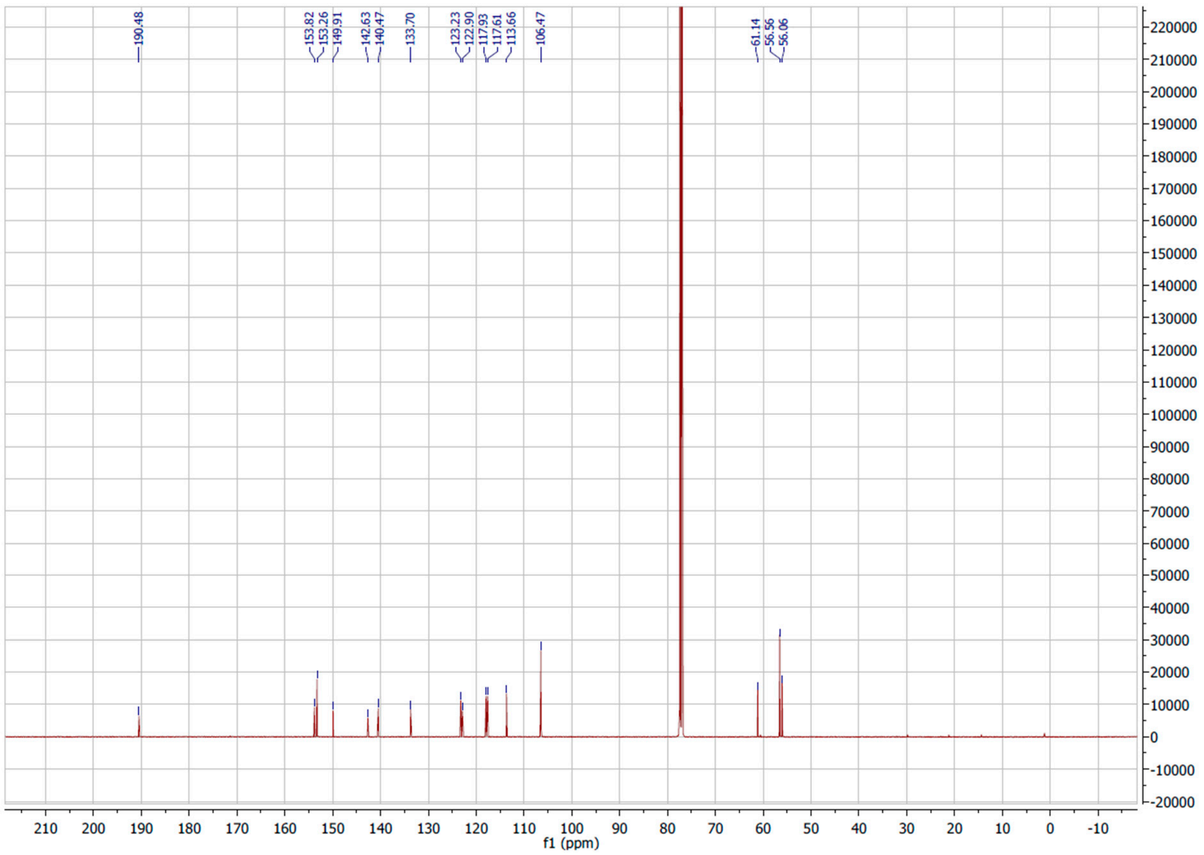

**Figure S12: $^{13}$C spectrum of chalcone 6**

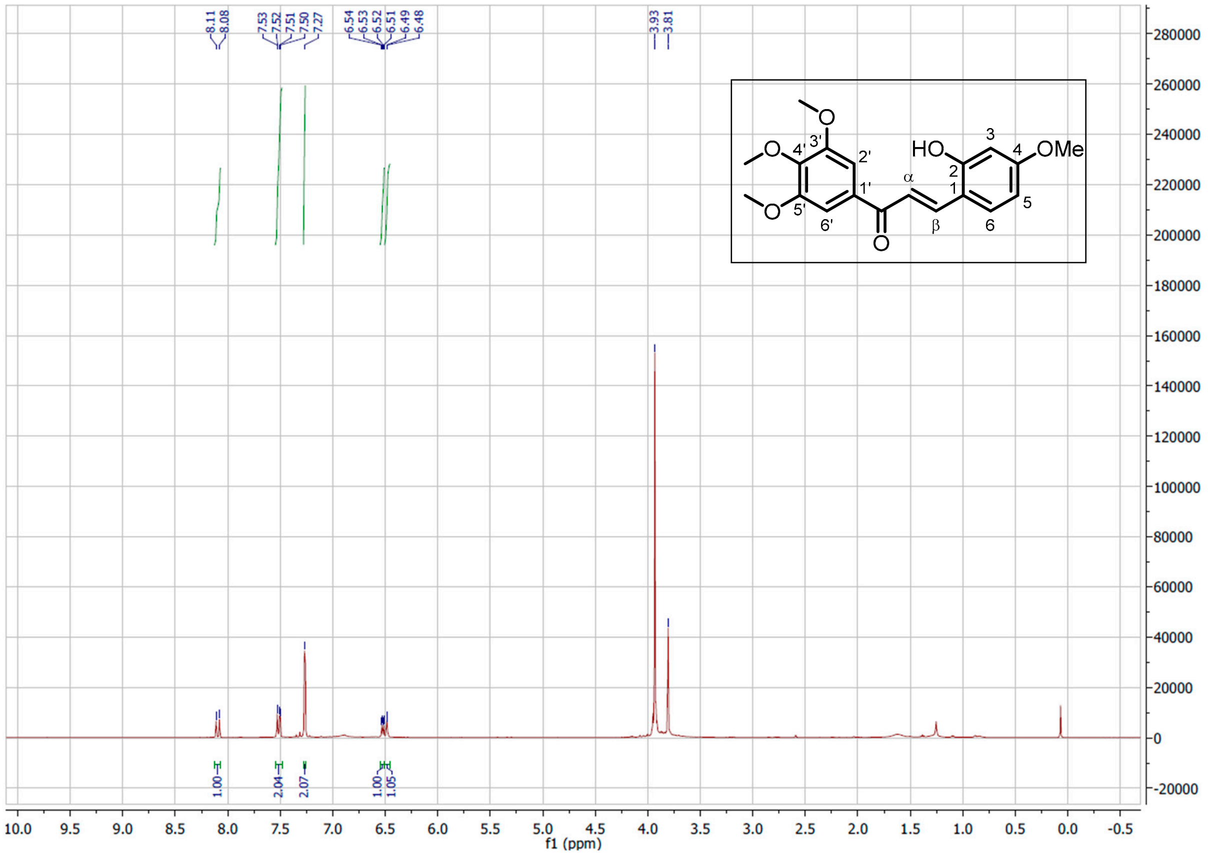


**Figure S13: $^{1}$H spectrum of chalcone 7**

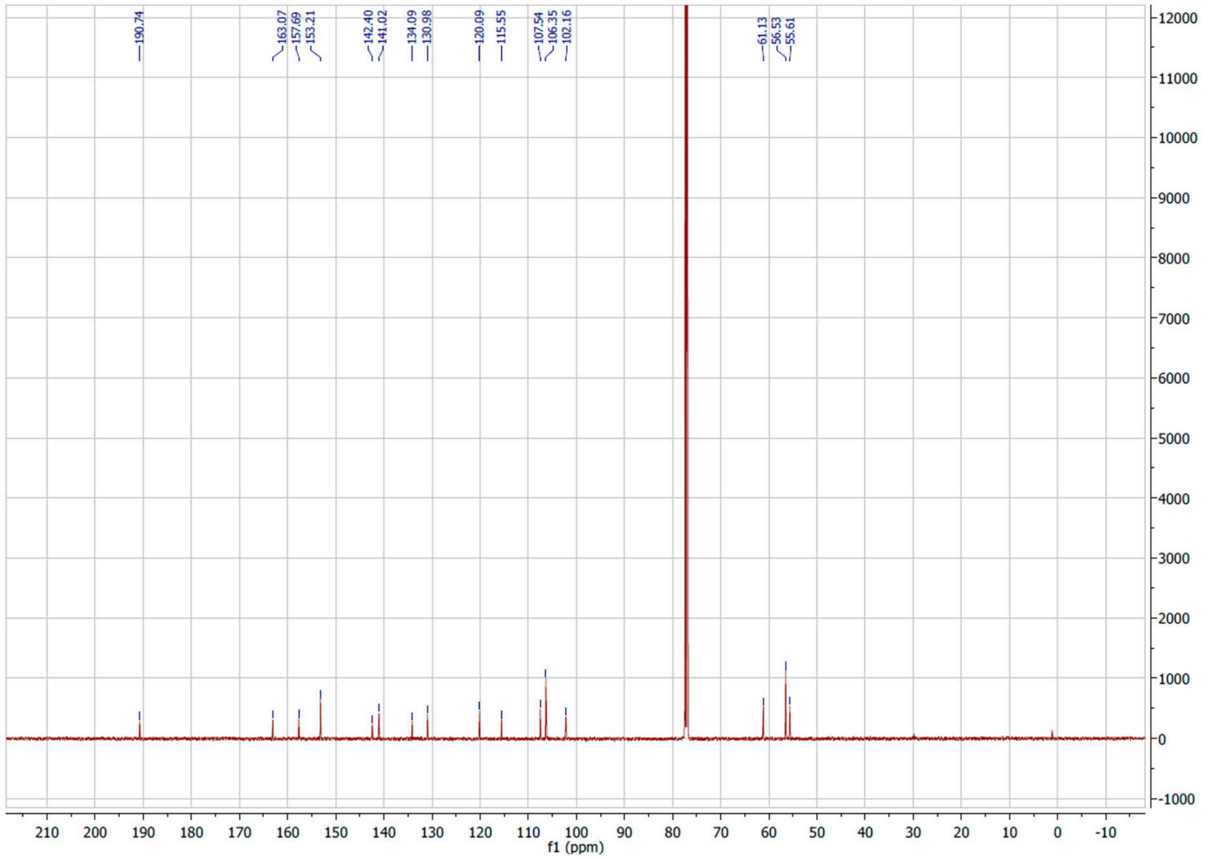


**Figure S14: $^{13}$C spectrum of chalcone 7**

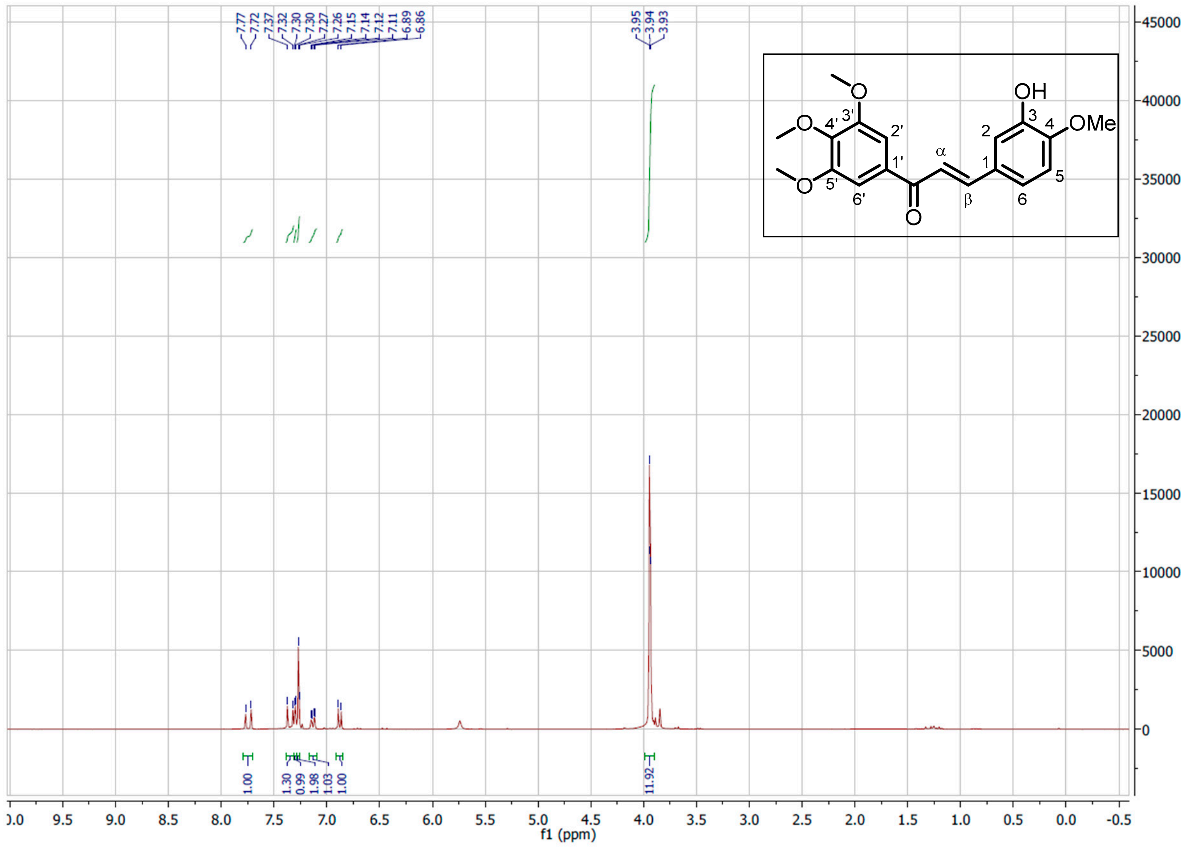

Figure S15: $^{1}$H spectrum of chalcone 8

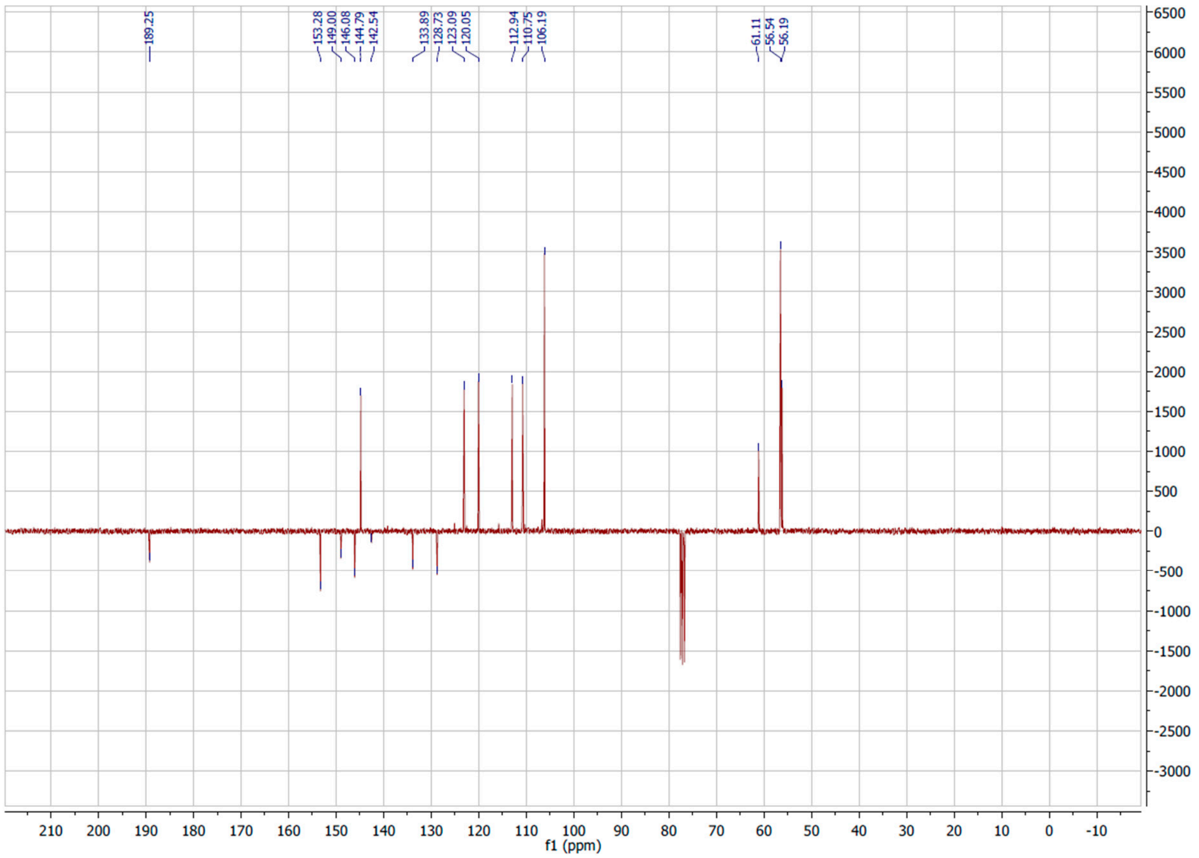

Figure S16: $^{13}$C spectrum of chalcone 8

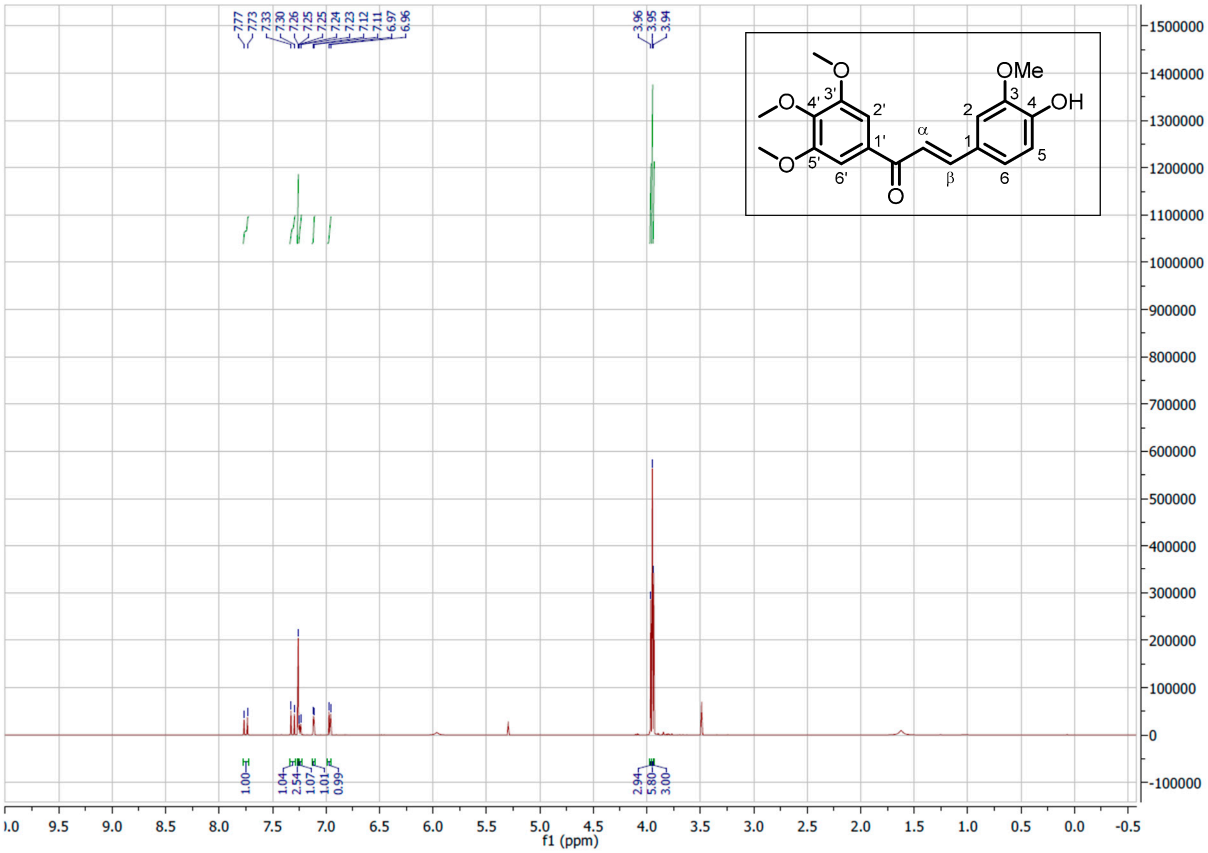

**Figure S17: $^{1}$H spectrum of chalcone 9**

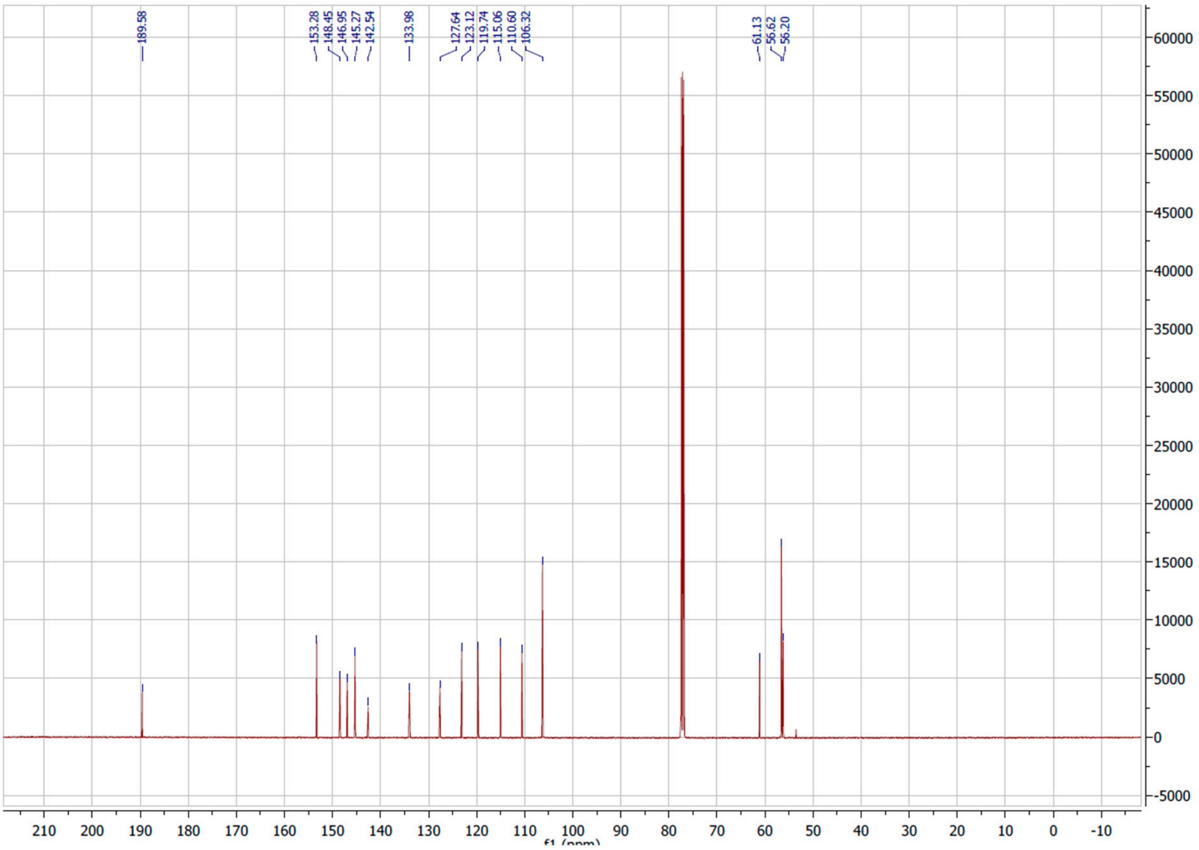

**Figure S18: $^{13}$C spectrum of chalcone 9**

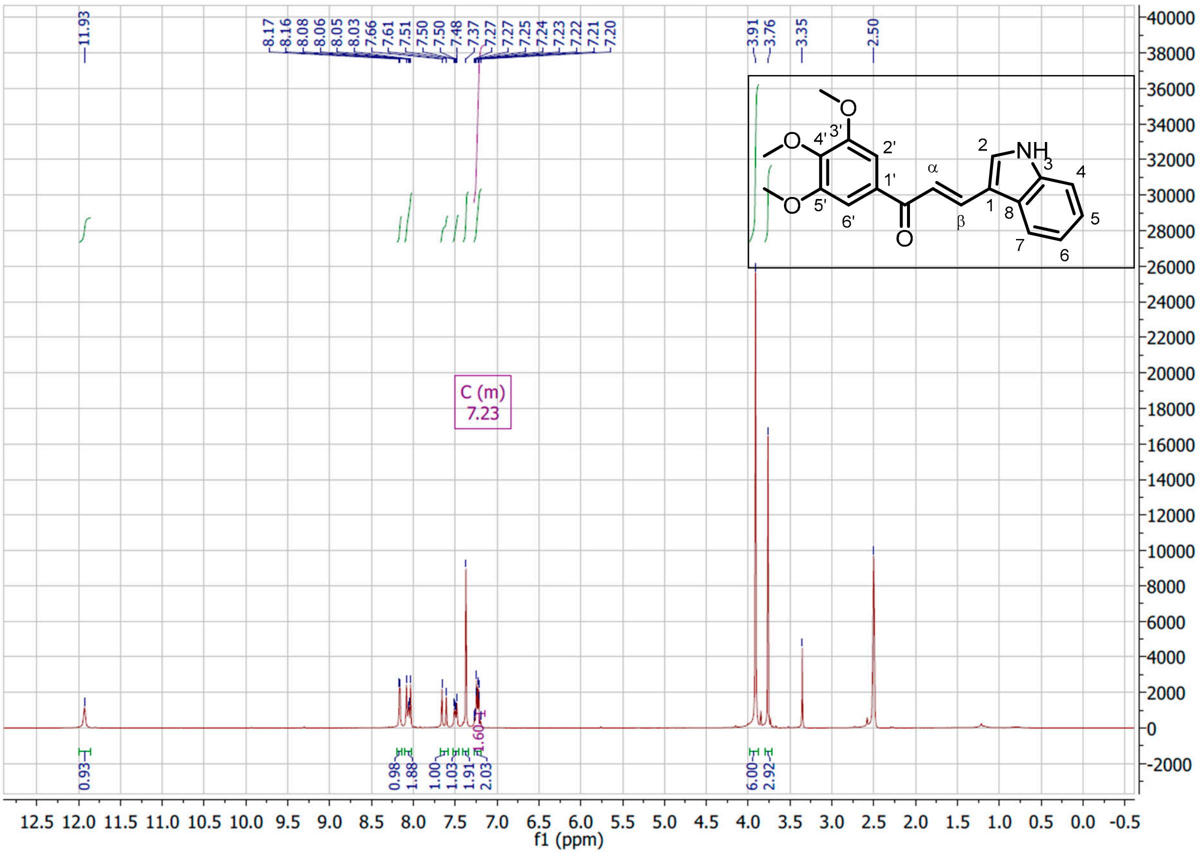

Figure S19: $^{1}$H spectrum of chalcone 10

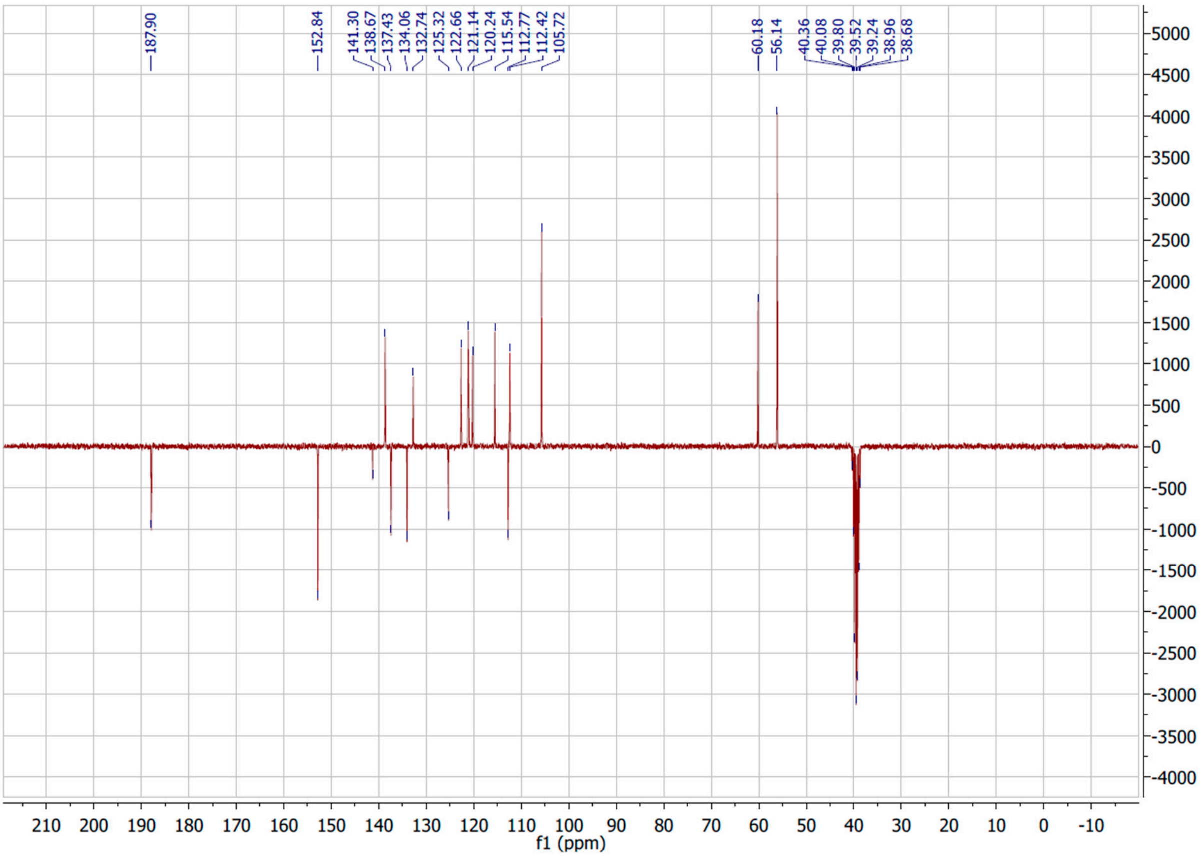

Figure S20: $^{13}$C spectrum of chalcone 10

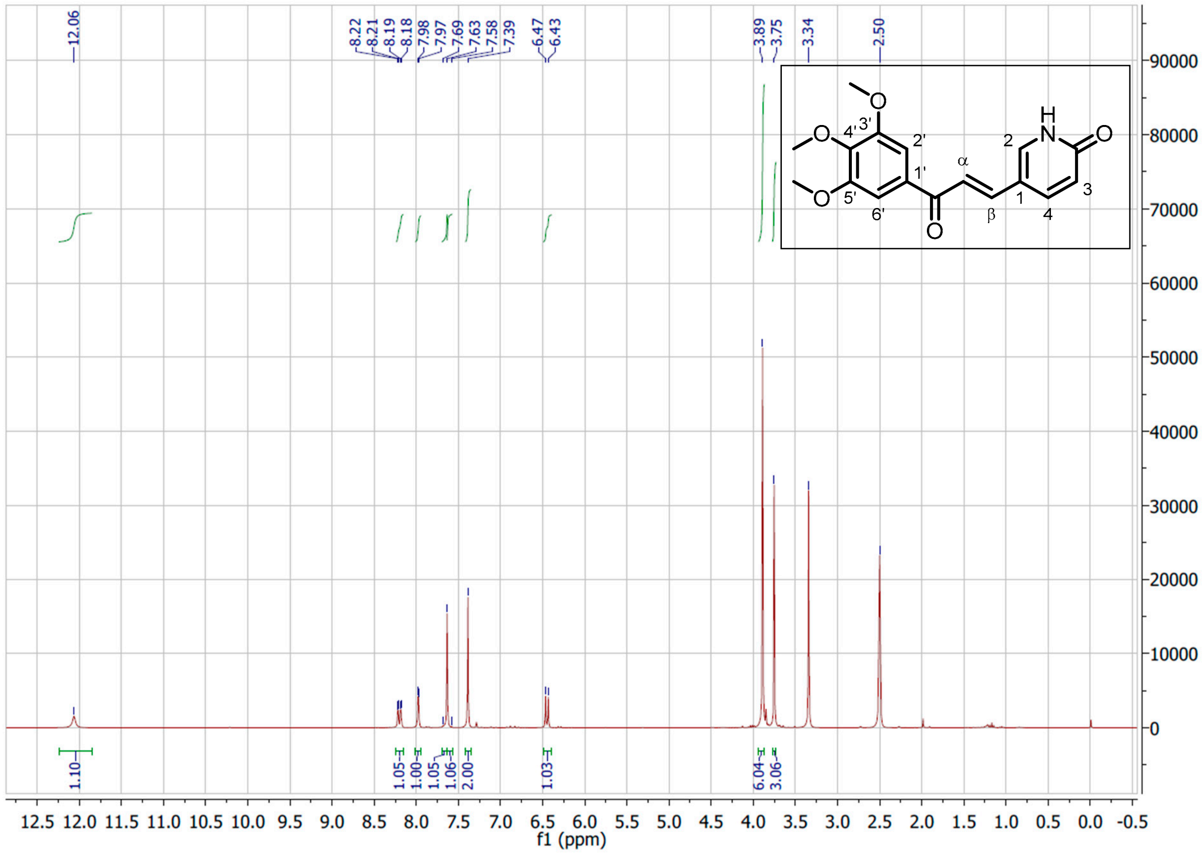


**Figure S21: $^1$H spectrum of chalcone 11**

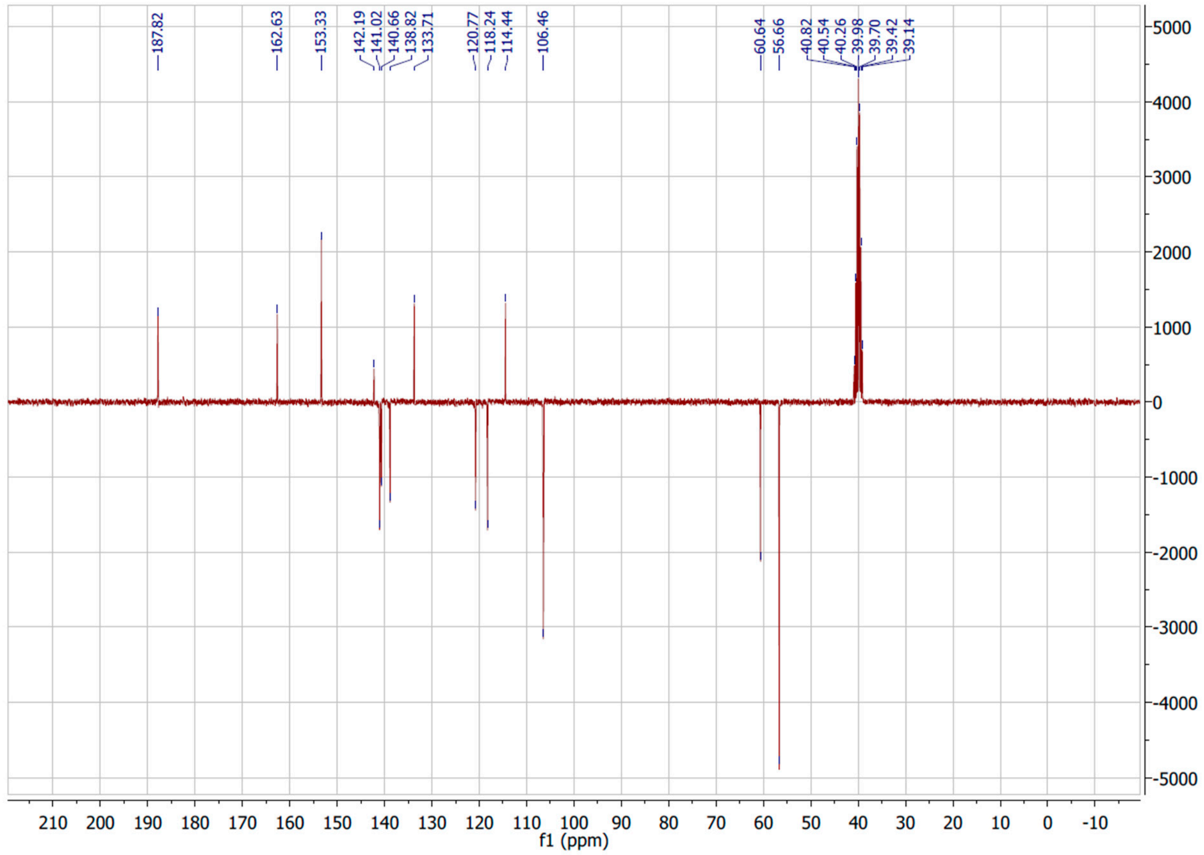

## Figure S22: ¹³C spectrum of chalcone 11

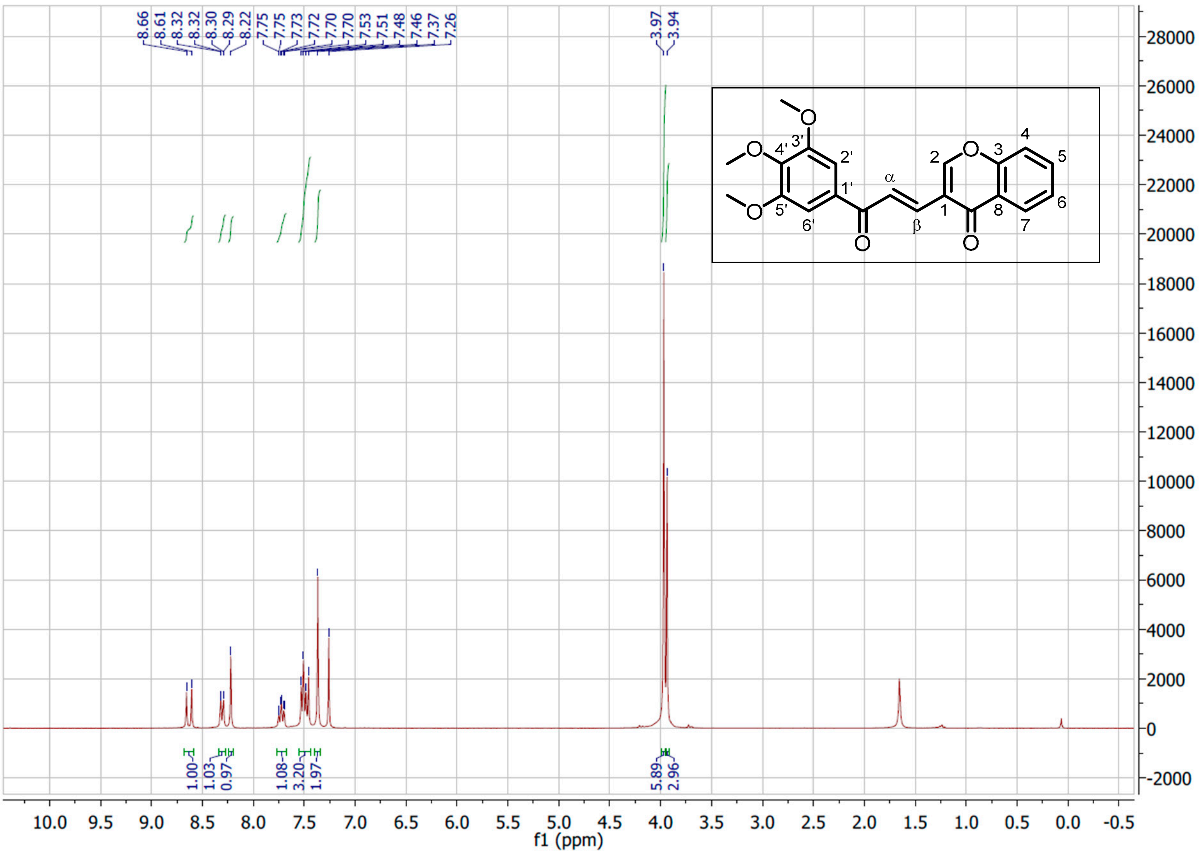


## Figure S23: ¹H spectrum of chalcone 12

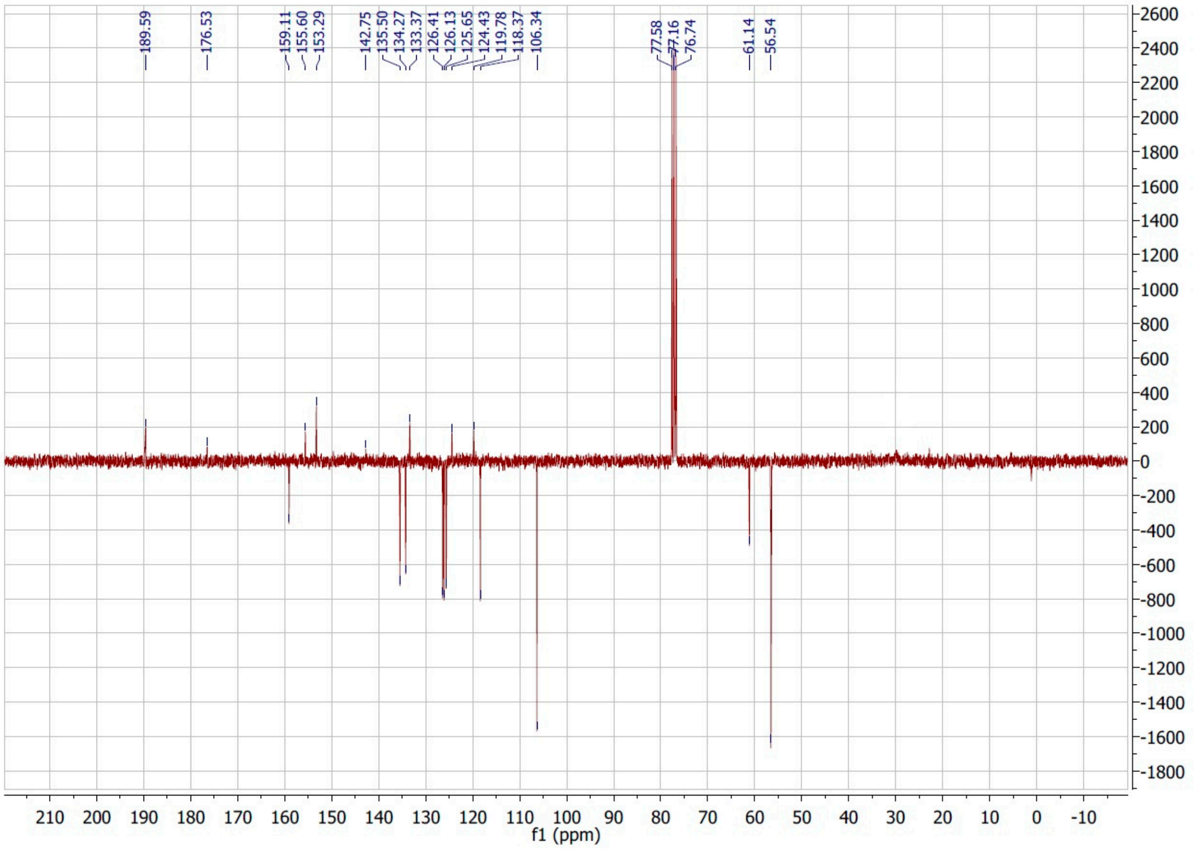

## Figure S24: <sup></sup>$^{13}$C spectrum of chalcone 12

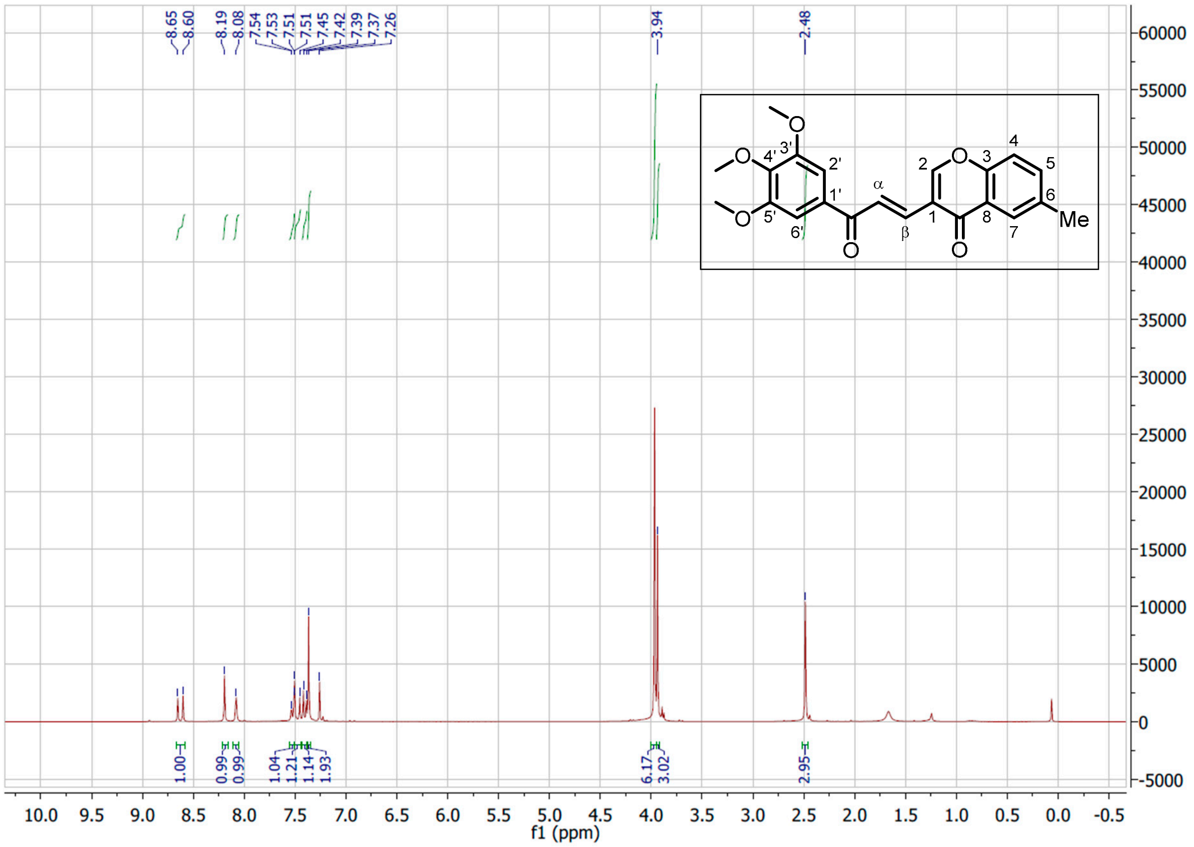

## Figure S25: $^{1}$H spectrum of chalcone 13

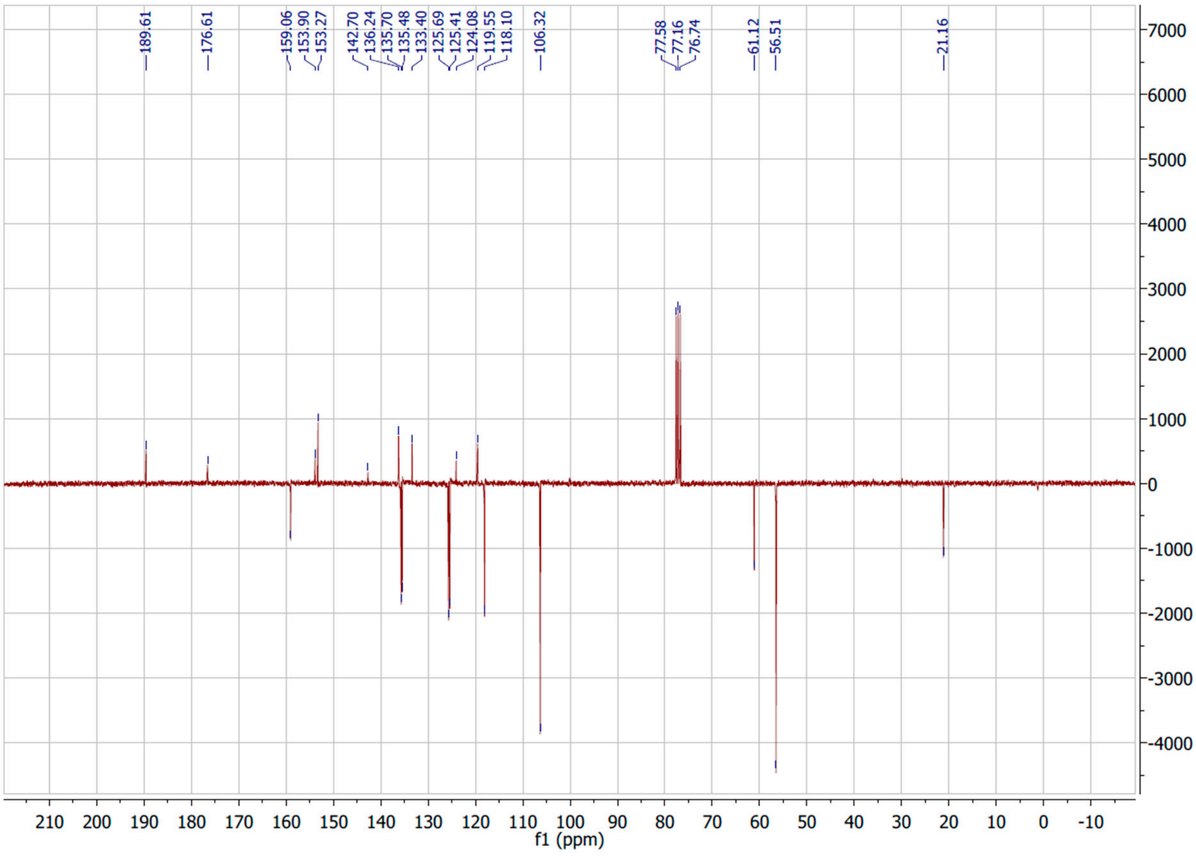

## Figure S26: $^{13}$C spectrum of chalcone 13

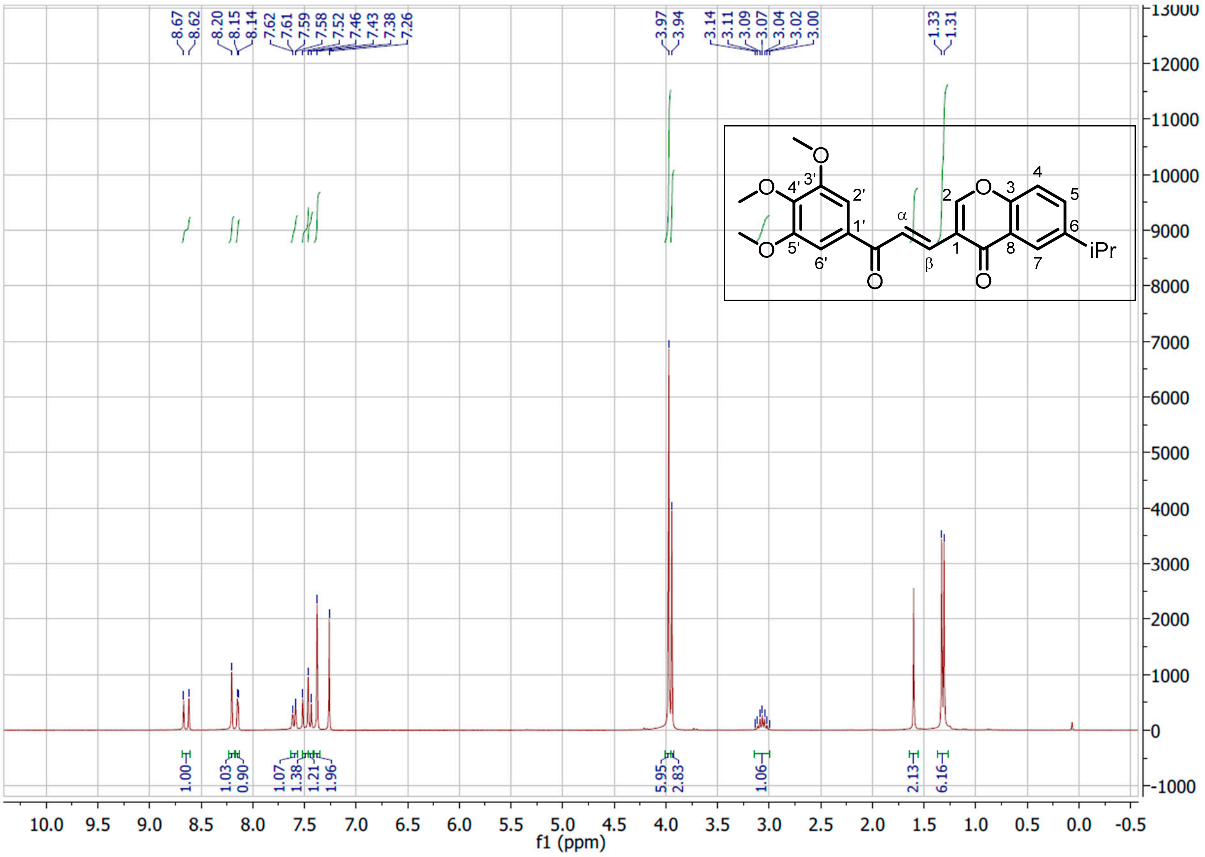


## Figure S27: $^{1}$H spectrum of chalcone 14

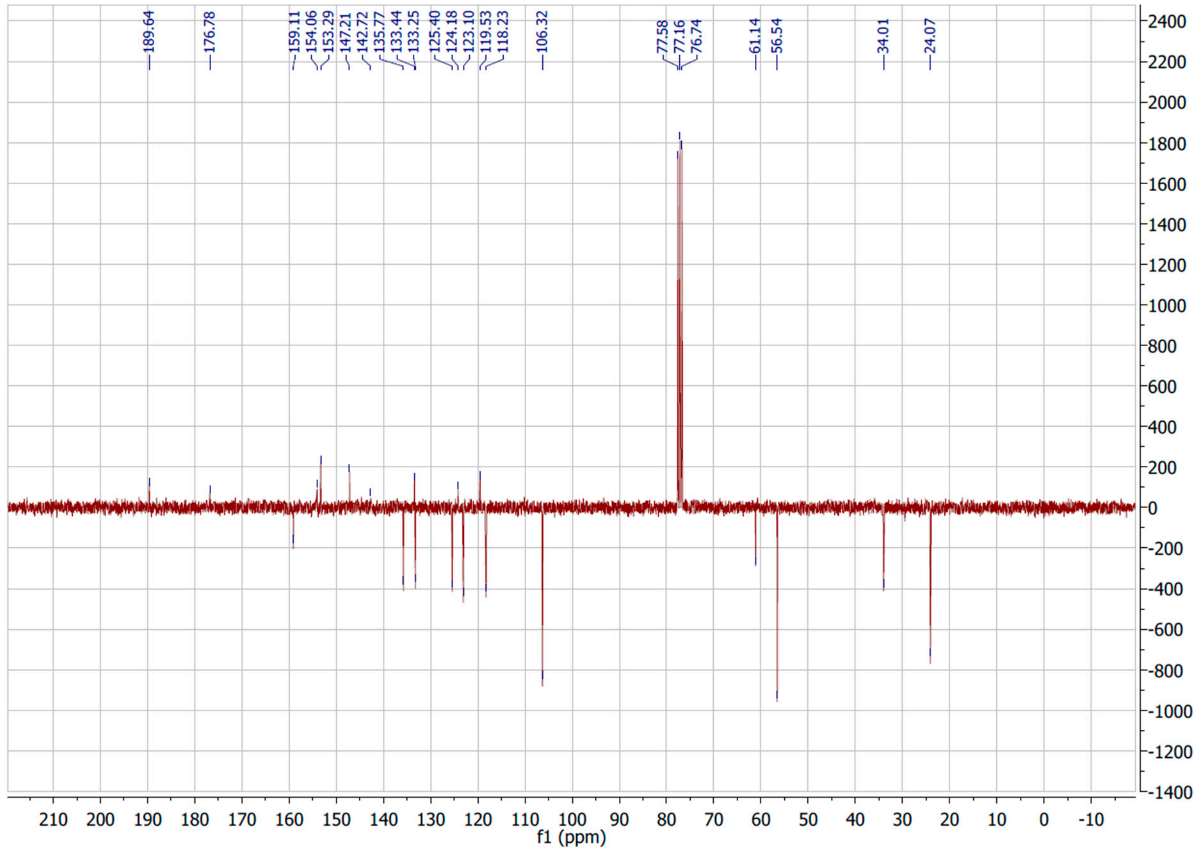

## Figure S28: $^{13}$C spectrum of chalcone 14

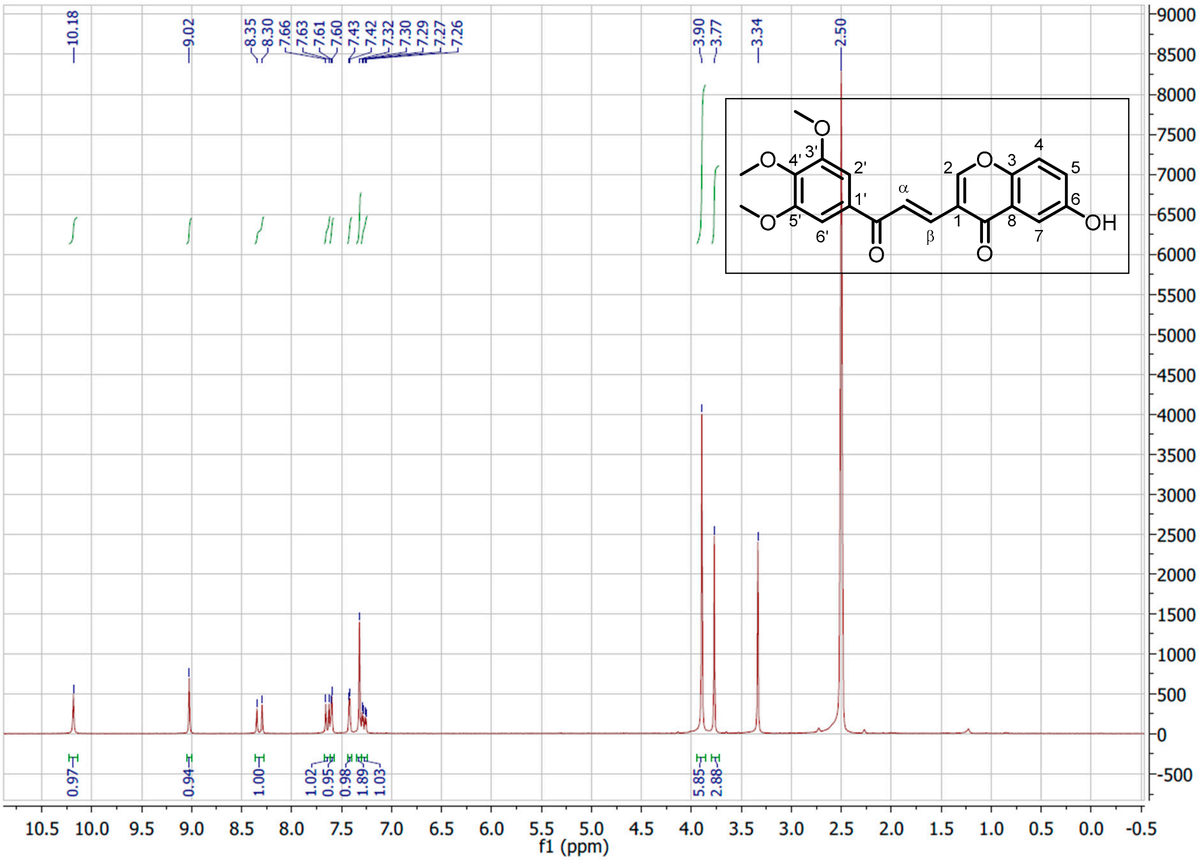


## Figure S29: $^{1}$H spectrum of chalcone 15

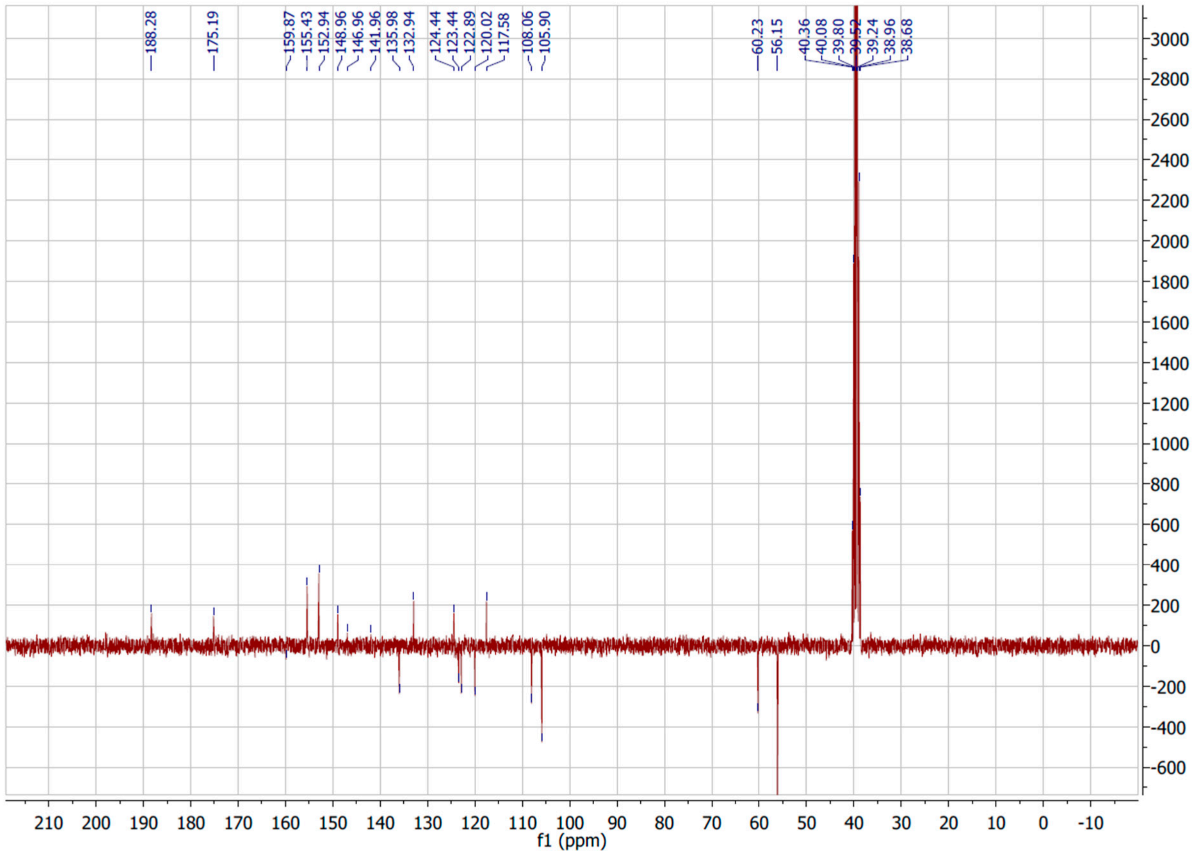

Figure S30: $^{13}C$ spectrum of chalcone 15

## HRMS analysis

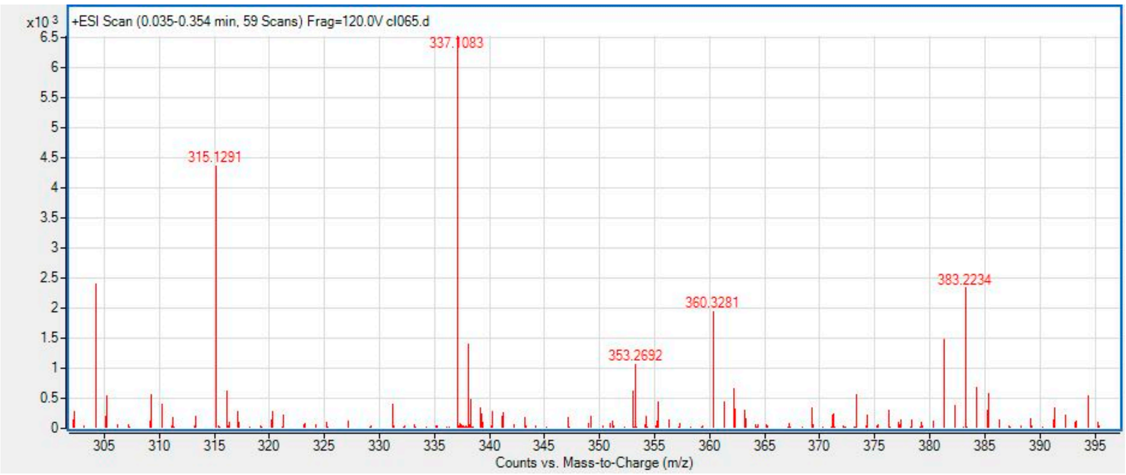


Figure S31. HRMS spectrum of chalcone 1

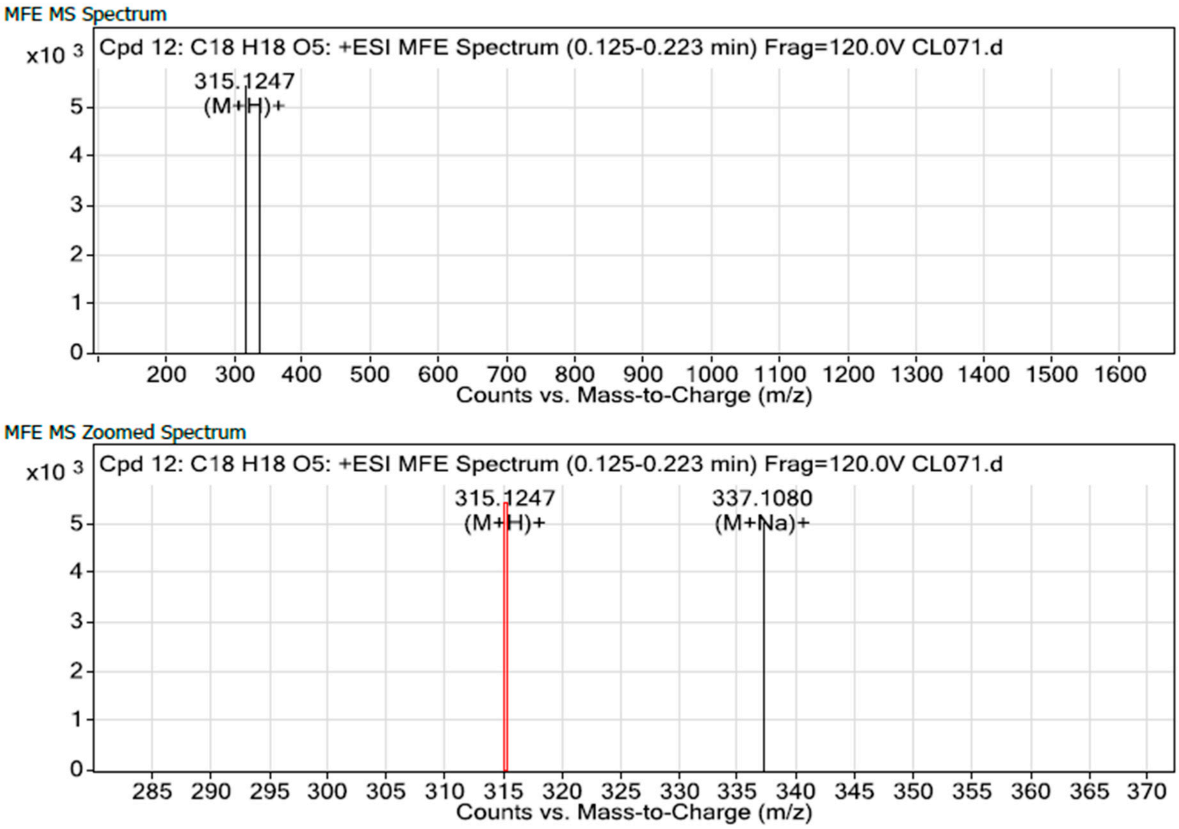


Figure S32. HRMS spectrum of chalcone 2

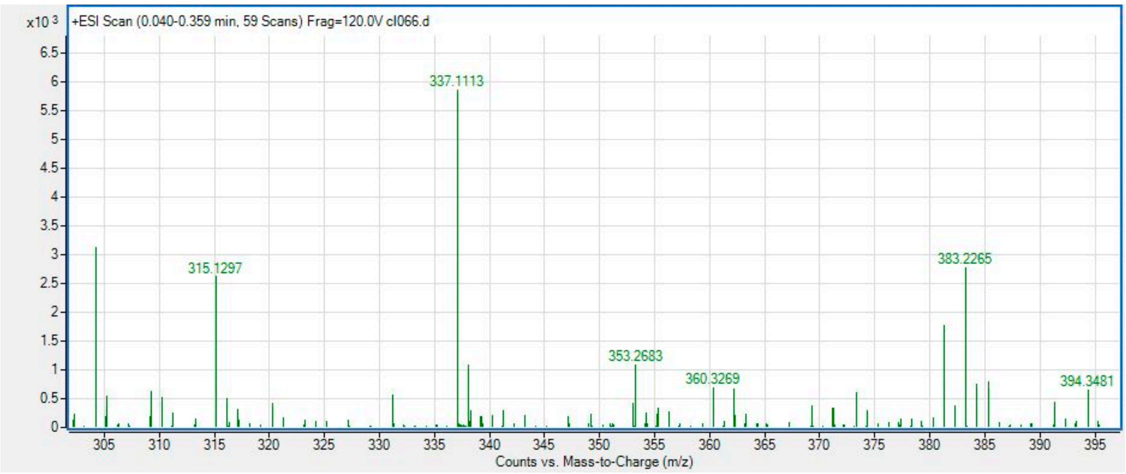


**Figure S33. HRMS spectrum of chalcone 3**

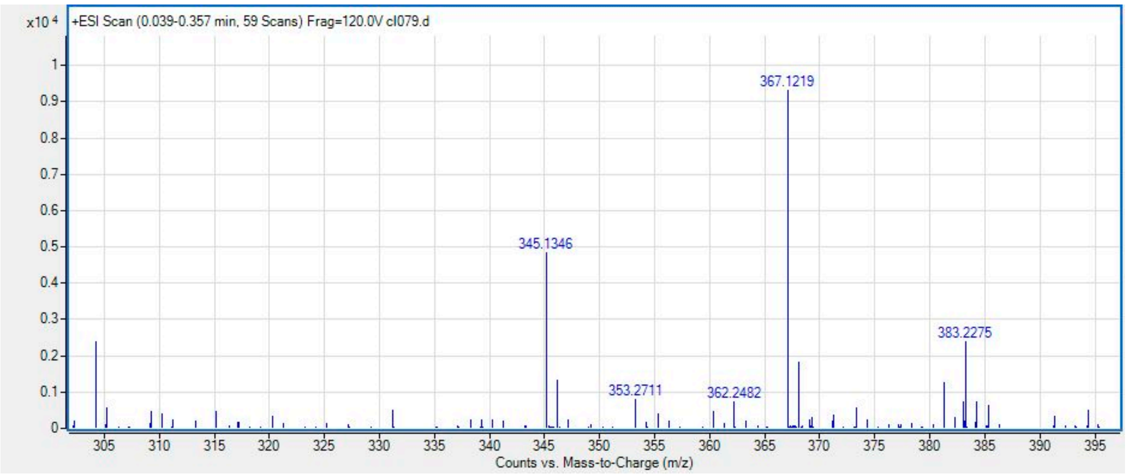


**Figure S34. HRMS spectrum of chalcone 4**

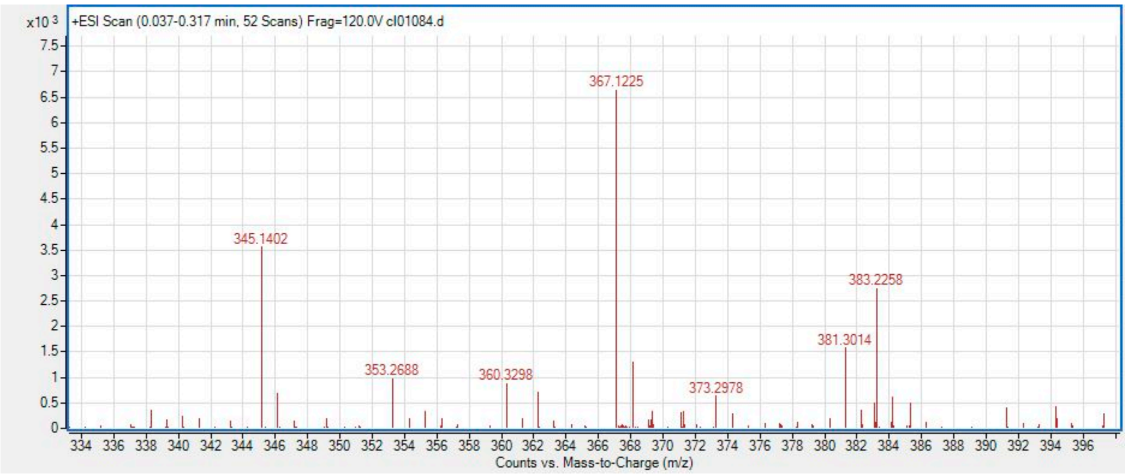


**Figure S35. HRMS spectrum of chalcone 5**

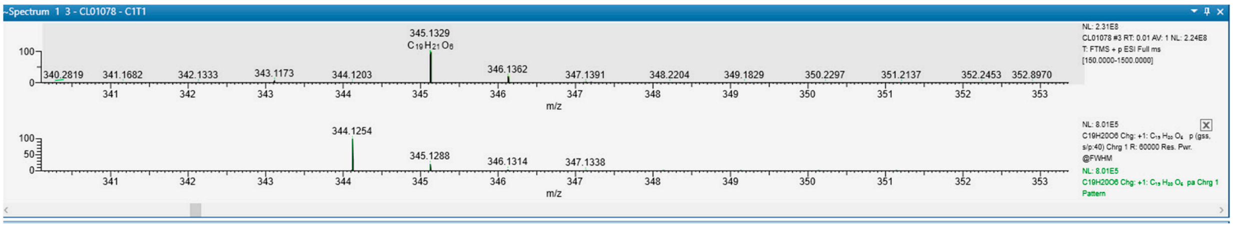


Figure S36. HRMS spectrum of chalcone 6

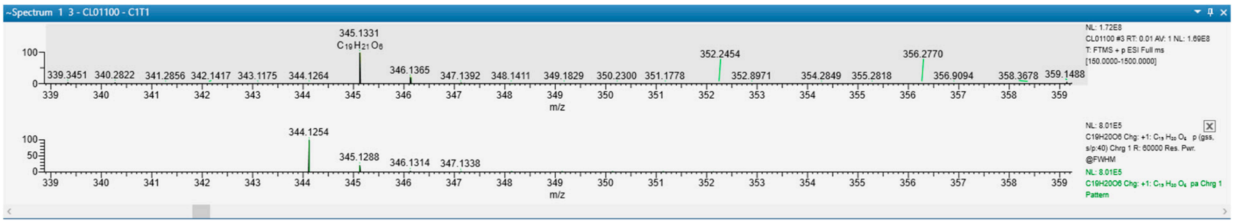


Figure S37. HRMS spectrum of chalcone 7

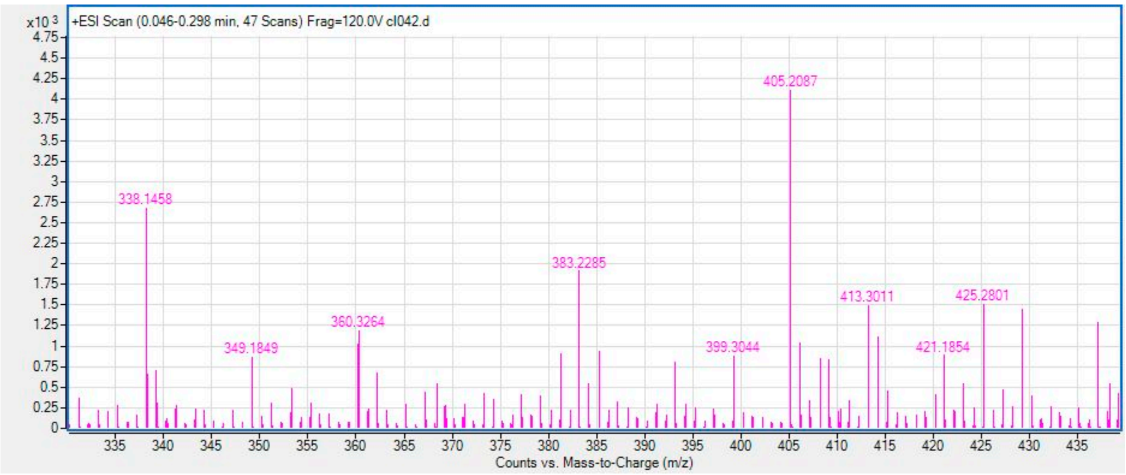


Figure S38. HRMS spectrum of chalcone 10

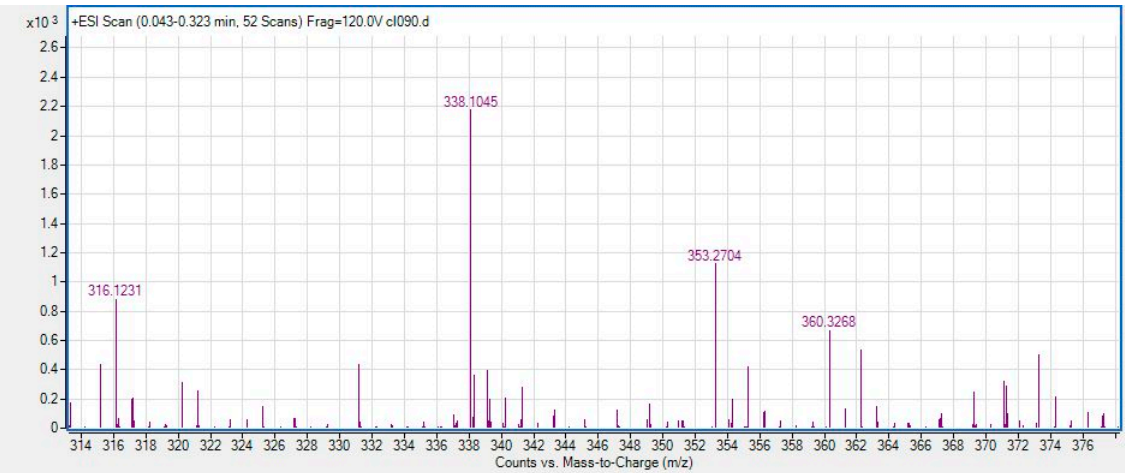


Figure S39. HRMS spectrum of chalcone 11

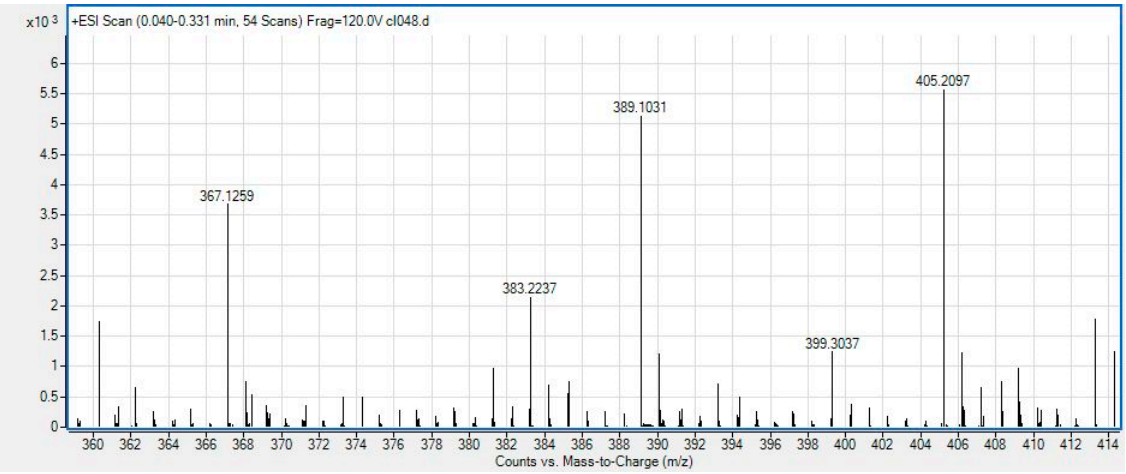


**Figure S40. HRMS spectrum of chalcone 12**

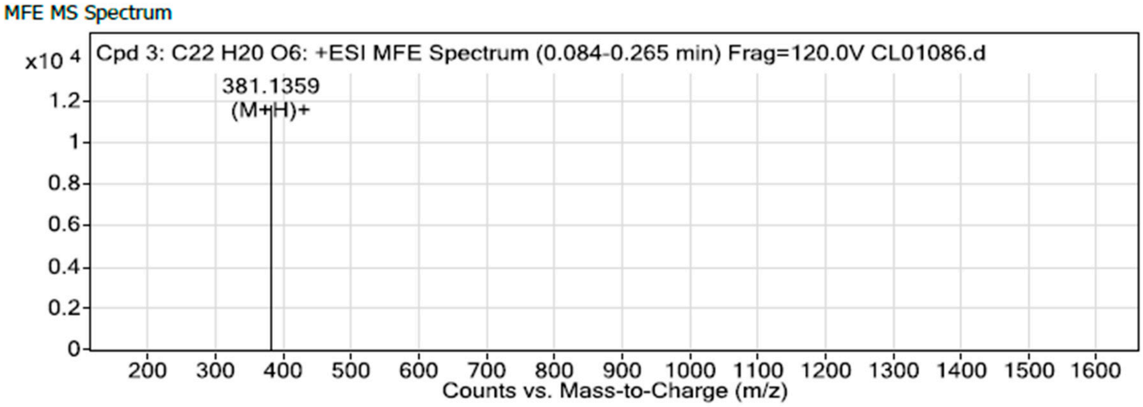


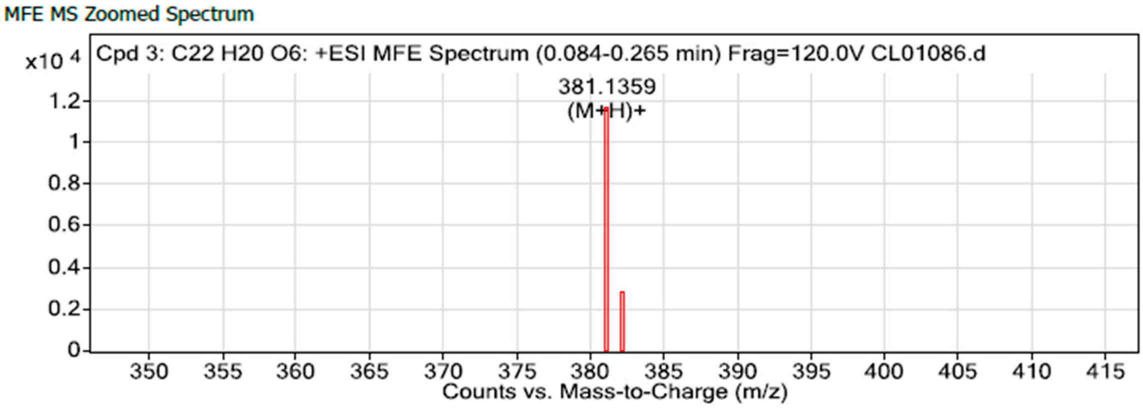

## Figure S41. HRMS spectrum of chalcone 13

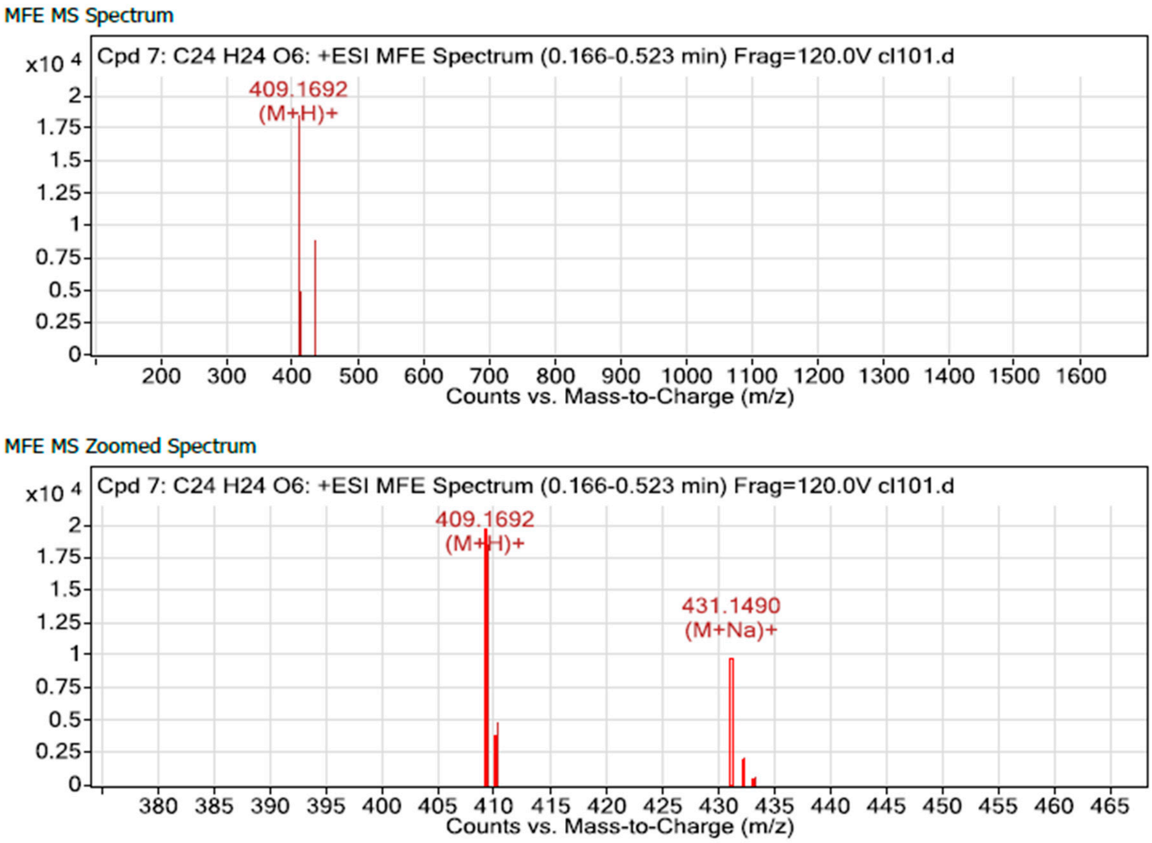


## Figure S42. HRMS spectrum of chalcone 14

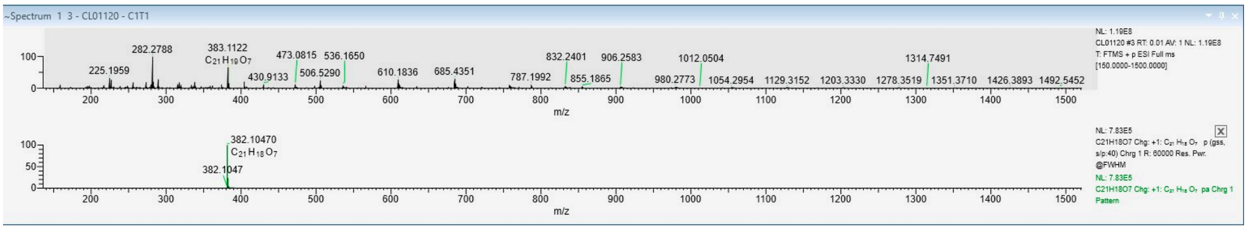


## Figure S43. HRMS spectrum of chalcone 15

## Protocols

### Protocol S1. Synthesis of 6-oxo-1,6-dihydropyridine-3-carbaldehyde:

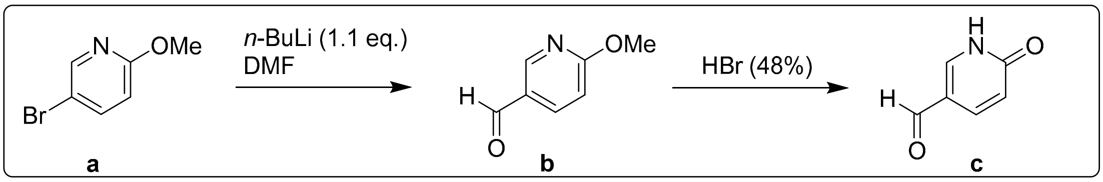


Butyllithium (12.7 mL, 2.2 M solution in hexanes, 27.9 mmol) was added dropwise to a solution of 2-methoxy-5-bromopyridine (**a**; 5 g, 26.6 mmol) in dry tetrahydrofuran (40 mL) at -78 °C and the suspension stirred at -78 °C for 45 min. *N,N*-Dimethylformamide (4.1 mL, 53.2 mmol) was then added, dropwise, and stirring continued for a further 90 min. The mixture was allowed to warm to room temperature and then it was poured into $NaHCO_3$ (aqueous saturated) and extracted with ether (3×50 mL). The combined organic extracts were dried over $MgSO_4$, and the solvent was removed at reduced pressure to give 3.26 g of 6-methoxy-3-pyridine-carboxaldehyde (**b**) as a pale yellow solid. The physical and spectral data were found to be identical to those published in the literature.

Then, a solution of 6-methoxy-3-pyridinecarboxaldehyde (**b**; 3.20 g, 23.3 mmol) was dissolved in HBr (48% aqueous, 45 mL), and the temperature of the heating bath was gradually raised to 150 °C over a period of 1 h. Gas evolution was observed at 90-110 °C. The dark red-brown solution was allowed to cool and concentrated to a light brown solid. 9 mL of MeOH and 4 mL of acetone were added, and the solution was washed with ether (3 × 40 mL). Then 10 mL of water (mL) was added and the pH adjusted to ca. 7 with $NaHCO_3$ (aqueous saturated). A small amount of brown solid was removed by filtration and the filtrate concentrated into a tan solid. This solid was taken into $CHCl_3$/MeOH (7:3) as solvent and filtered through a silica plug. Removal of the solvent at reduced pressure gave 6-oxo-1,6-dihydropyridine-3-carbaldehyde (**c**; 1.07 g, 37%). The physical and spectral data were found to be identical to those published in the literature.

### Protocol S2. General procedure for the synthesis of 4-oxo-4*H*-chromene-3-carbaldehydes:

The 4-oxo-4*H*-chromene-3-carbaldehydes (or 3-formylchromones) used as starting materials were prepared by an improved method according to Nohara's procedure. Over an ice bath containing a stirred solution of appropriate 2-hydroxyacetophenones in DMF, the $POCl_3$ (5 equiv.) was added slowly through a dropping funnel and stirring was continued at 0 °C until no gas emissions were observed. Then the mixture was kept at room temperature until the starting material had been completely consumed (TLC analysis). The reaction was hydrolyzed by pouring it into crushed ice. After stirring for a few minutes, the formed precipitate was filtered, washed with water, and dried to yield pure desired chromones.

## Cell viability

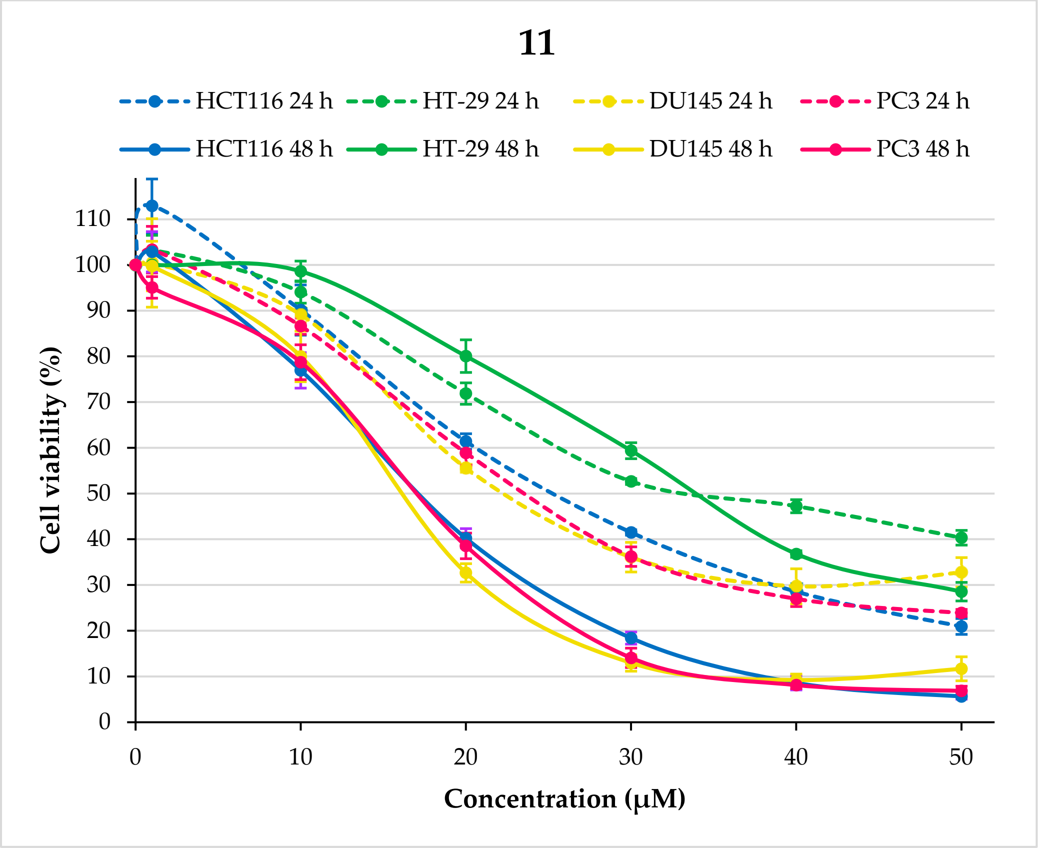


**Figure S44. Effect of chalcone 11 on cell viability in human CRC (HCT116 and HT-29) and human PCa (DU145 and PC3) cells at 24 and 48 h.**

Results were expressed as percentage of viability compared to control. Results were presented as means ± SEM of three independent experiments.

## Trypan blue dye exclusion method

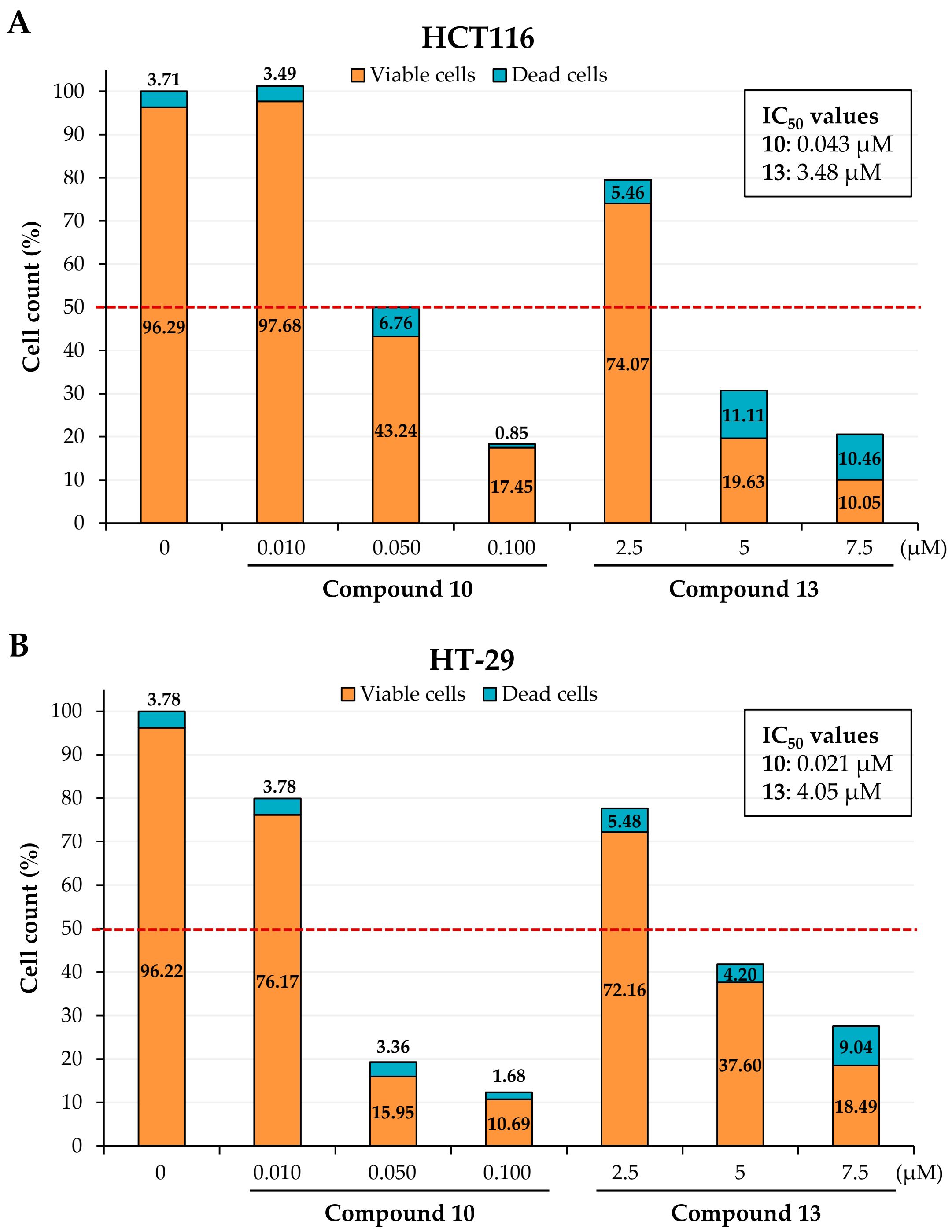


**Figure S45. Cell count at 48 h (viable and dead cells) relative to the concentration of compounds 10 and 13 for human colorectal (HCT116 and HT-29) cancer cells.**

The control was normalized to 100% including viable and dead cells. Results were expressed as percentage of cells compared to control.

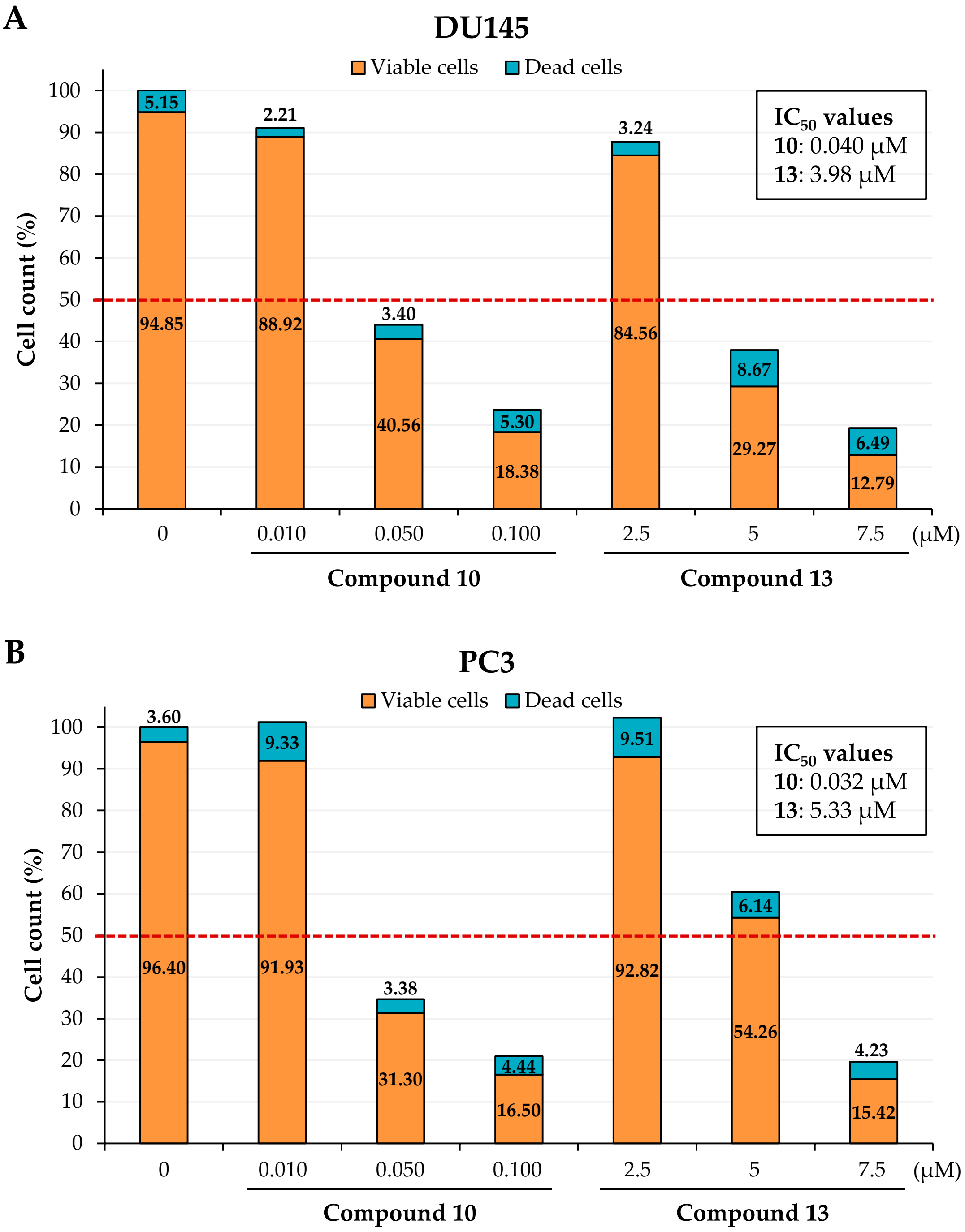


**Figure S46. Cell count at 48 h (viable and dead cells) relative to the concentration of compounds 10 and 13 for human prostatic (DU145 and PC3) cancer cells.**

The control was normalized to 100% including viable and dead cells. Results were expressed as percentage of cells compared to control.
